# *OlCHR,* encoding a chromatin remodeling factor, is a killer causing hybrid sterility between rice species *Oryza sativa* and *O. longistaminata*

**DOI:** 10.1016/j.isci.2024.109761

**Published:** 2024-04-17

**Authors:** Zin Mar Myint, Yohei Koide, Wakana Takanishi, Tomohito Ikegaya, Choi Kwan, Kiwamu Hikichi, Yoshiki Tokuyama, Shuhei Okada, Kazumitsu Onishi, Ryo Ishikawa, Daisuke Fujita, Yoshiyuki Yamagata, Hideo Matsumura, Yuji Kishima, Akira Kanazawa

**Affiliations:** 1 Research Faculty of Agriculture, Hokkaido University, Sapporo, Japan; 2 National Agriculture and Food Research Organization, Tsukuba, Japan; 3 Obihiro University of Agriculture and Veterinary Medicine, Obihiro, Japan; 4 Graduate School of Agricultural Science, Kobe University, Kobe, Japan; 5 Faculty of Agriculture, Saga University, Saga, Japan; 6 Research Faculty of Agriculture, Kyushu University, Fukuoka, Japan; 7 Gene Research Centre, Shinshu University, Ueda, Japan

**Keywords:** Plant genetics, Plant biology, Plant evolution

## Abstract

The genetic mechanisms of reproductive isolation have been widely investigated within Asian cultivated rice (*Oryza sativa*); however, relevant genes between diverged species have been in sighted rather less. Herein, a gene showing selfish behavior was discovered in hybrids between the distantly related rice species *Oryza longistaminata* and *O. sativa*. The selfish allele *S13*^*l*^ in the *S13* locus impaired male fertility, discriminately eliminating pollens containing the allele *S13*^*s*^ from *O. sativa* in heterozygotes (*S13*^*s*^/*S13*^*l*^). Genetic analysis revealed that a gene encoding a chromatin-remodeling factor (CHR) is involved in this phenomenon and a variety of *O. sativa* owns the truncated gene *OsCHR745*, whereas its homologue *OlCHR* has a complete structure in *O. longistaminata*. CRISPR-Cas9-mediated loss of function mutants restored fertility in hybrids. African cultivated rice, which naturally lacks the *OlCHR* homologue, is compatible with both *S13*^*s*^ and *S13*^*l*^ carriers. These results suggest that *OlCHR* is a Killer gene, which leads to reproductive isolation.

## Highlights

• *OlCHR* is a Killer element that is necessary for a transmission advantage
• CRISPR-Cas9-induced knockout mutants for *OlCHR* break the sterility and *m*TRD
• A naturally occurring lack of the homologue of *OlCHR* produces a neutral allele

## Graphical abstract

**Figure:**
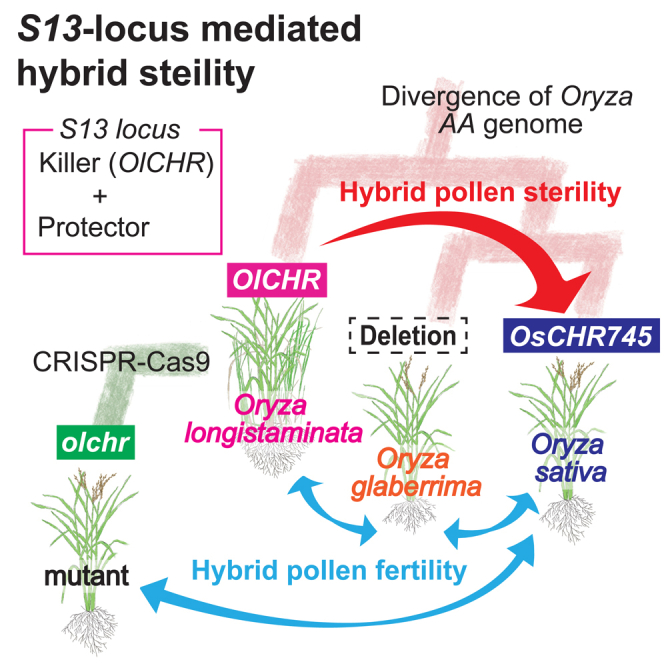

## Introduction

Reproductive isolation interferes with gene exchange between two biological species.^1^^,^^2^^,^^3^^,^^4^ One of the fundamental questions in biology is understanding the origin of species. Consequently, the evolutionary processes of genes leading to reproductive isolation are of great interest to biologists.^5^^,^^6^ Among various reproductive isolation mechanisms, intrinsic postzygotic isolation barriers, such as hybrid inviability, hybrid sterility, and hybrid breakdown have been widely investigated.^7^ Because genes causing hybrid incompatibility reduce fitness in zygotes or gametes at birth, how such genes evolved and became fixed within a species without falling into fitness valleys^7^ has been a long-debated issue. Internal genetic conflicts caused by selfish genetic elements are considered one of the evolutionary forces causing hybrid incompatibility.^8^^,^^9^ Selfish genetic elements may be rapidly fixed in a population due to their “selfish nature” but may become suppressed to reduce or eliminate their deleterious effects.^10^^,^^11^ If this population hybridizes with a population which has not experienced the conflict, hybrid incompatibilities can occur when the selfish genetic element segregates away from the suppressor, unleashing hybrid conflict.^8^^,^^12^

Selfish genetic elements causing hybrid incompatibilities have been frequently reported in plant species, particularly in rice and its relatives from the *Oryza* genus. This genus comprises two cultivated rice species (*O. sativa* and *Oryza glaberrima*) and five wild species (*Oryza rufipogon*, *Oryza barthii*, *Oryza glumaepatula*, *Oryza meridionalis*, and *Oryza longistaminata*), all sharing the same AA genome type (here, we refer to this species group as *Oryza* AA genome species in accordance with Mochizuki et al.^13^ and Cheng et al.^14^). Extensive research has focused on hybrid incompatibilities within Asian cultivated rice (*O. sativa*) varieties, with over 50 genes associated with hybrid incompatibilities being reported.^15^^,^^16^^,^^17^^,^^18^ Interestingly, most of these reported genes are related to hybrid sterility and exhibit selfish genetic actions. In these cases, a gamete carrying a specific allele preferentially transmits to the next generation, often due to abortion in gametes with an alternative allele in heterozygosity.^18^ This preferential gamete abortion results in transmission ratio distortion (TRD) in the subsequent generation.^18^^,^^19^^,^^20^ The selective abnormality occurs in either male (*m*TRD)/female (*f*TRD) -gametes or sex-independently (*si*TRD).^18^ The abundance of these hybrid sterility genes with selfish nature, that are also known as one-locus sporogametophytic hybrid sterility genes, and the prevalence of TRD underscore their evolutionary significance in rice species. However, our understanding of hybrid incompatibilities in distantly related *Oryza* AA genome species remains limited.

Recent studies have revealed the genetic characteristics of selfishness in hybrid sterility genes in rice.^21^^,^^22^^,^^23^^,^^24^^,^^25^^,^^26^^,^^27^^,^^28^^,^^29^ These genes typically consist of two tightly linked components, which function as Killers and Protectors (or Toxins and Antidotes), respectively.^22^^,^^24^^,^^26^^,^^27^^,^^29^ This arrangement is based on the two-component hybrid sterility system originally described for the *t*-haplotype in *Mus musculus*.^30^ For example, in the recently characterized male gamete sterility locus *RHS12*, there are two genes (*iORF3/DUYAO* and *iORF4/JIEYAO*). *DUYAO* encodes a Toxin that interacts with *OsCOX11*, leading to mitochondrial malfunction and cytotoxicity. Meanwhile, *JIEYAO* encodes an Antidote that protects male gametes carrying it by inhibiting the DUYAO–OsCOX11 interaction.^27^ This Killer–Protector system has also been found in the *S5* locus-mediated hybrid sterility in Asian cultivated rice.^22^ Another genetic mechanism for hybrid sterility genes with selfish nature in this species involves a Killer–Responder system (a concept originally discussed in the *SD* system in *Drosophila melanogaster*; Merrill et al.^31^). For example, the hybrid sterility locus *Sa* comprises two adjacent genes, *SaM* (*SaM*+ and *SaM*− alleles) and *SaF* (the *SaF*− allele). In hybrid plants, the *SaM*+ and *SaF*+ proteins together form a Killer that targets pollen inheriting the *SaM−* (Responder) locus (Long et al.^21^; reviewed by Nunes et al.^32^).

Although these studies have indicated the widespread presence of hybrid sterility genes with Killer–Protector or Killer–Responder functions within Asian cultivated rice, it remains unclear whether such genetic elements are involved in hybrid sterility among *Oryza* AA genome species. In interspecific crosses between *Oryza* AA genome species, more pronounced pollen and seed sterility have been observed than in intraspecific crosses between Asian cultivated rice varieties.^33^ Recent molecular genetics approaches have identified causative genes for hybrid sterility among *Oryza* AA genome species.^23^^,^^24^^,^^25^^,^^26^^,^^29^^,^^34^ Among them, three loci (*S1*, *qHMS7* and *qHMS1*) are involved in hybrid sterility causing TRD between *Oryza* AA genome species. The *S1* locus, responsible for hybrid sterility between *O. sativa* and *O. glaberrima* (African cultivated rice species), consists of three tightly linked genes: *OgTPR1*, *SSP* (*S1A6*), and *S1A4*.^23^^,^^24^^,^^26^ All three are essential for its Killer function, with *OgTPR1* acting as a Protector. The *qHMS7* locus leads to hybrid sterility between *O. sativa* and *O. meridionalis* (Australian wild rice species) and comprises two tightly linked genes, ORF2 and ORF3.^25^ ORF2 encodes a toxic genetic element that induces pollen abortion in a sporophytic manner, whereas ORF3 encodes an Antidote that protects pollen in a gametophytic manner.^25^ Additionally, *qHMS1* is another hybrid sterility locus that adopts Killer-Protector (also termed as Toxin-Antidote) system between *O. sativa* and *O. meridionalis* found by You et al.^29^ *ORF3* (*HPT*) sporophytically encodes the indiscriminate toxic action while *ORF5* (*HPA*) gametophytically rescues only pollen with its presence. Comprising of functional toxin and antidote genes in *qHMS1-Mer*, it causes the *m*TRD and takes the transmission advantage over *qHMS1-D*. These studies indicate the existence of a Killer–Protector system not only within Asian cultivated rice species but also between *Oryza* species. However, to fully understand the evolutionary significance of the abundance of such hybrid sterility genes and TRD, it is necessary to identify the hybrid sterility genes active in other species pairs.

Hybrid sterility among species poses a significant barrier to gene flow and is considered a major obstacle for interspecific hybrid breeding.^35^^,^^36^ Recent studies have demonstrated that mutagenesis of Killer function genes within the hybrid sterility locus can yield alleles that do not induce sterility in heterozygotes.^23^^,^^24^^,^^37^ Therefore, identifying the causative genes for hybrid sterility and generating hybrid-compatible alleles through mutagenesis are potential approaches for future interspecific hybrid breeding programs.

Herein, we discovered the hybrid sterility locus *S13* in the distantly related *Oryza* AA genome species pair—*O. sativa* and *O. longistaminata*. The latter is a perennial-type wild rice distributed in Africa.^38^^,^^39^ This species exhibits unique characteristics such as rhizomatousness and partial self-incompatibility, which are traits not observed in other *Oryza* AA genome species.^39^^,^^40^ Several studies employing genome-wide molecular markers have shown that *O. longistaminata* is one of the most divergent species from *O. sativa* in the *Oryza* AA genome species.^38^^,^^41^^,^^42^ Severe pollen and embryo sac sterility have been observed in hybrids between *O. longistaminata* and *O. sativa*.^33^ To date, three QTLs (*qpsf6*, *S44*(t), *S40*) associated with hybrid sterility between *O. sativa* and *O. longistaminata* have been reported.^43^^,^^44^^,^^45^ However, none of these genes have been cloned; therefore, the molecular basis of hybrid sterility in these two species remains unknown. In 1995, a locus, *S13*, causing hybrid sterility in these two species has been registered by Prof. Sano,^46^^,^^47^ though its causative gene has been uninvestigated. In the present study, we use the same materials used by Prof. Sano and report the presence of the selfish allele in the *S13* locus, which causes sterility in hybrids between *O. sativa* and *O. longistaminata*. High-resolution mapping, genome analysis, and CRISPR-Cas9-mediated mutagenesis revealed the causative gene of the *S13* locus-mediated hybrid sterility. The causative gene was located in the chromosomal position identical to *HPT* gene in the *qHMS1* locus.^29^ The findings suggest the presence of a hybrid sterility gene causing TRD and a Killer system in a distantly related pair of *Oryza* AA genome species.

## Results

### The *S13* locus induces hybrid male sterility and male-specific TRD

The pollen fertility of the F_1_ between T65 (*O. sativa*) and W1618 (*O. longistaminata*, Figure S1) was approximately 2.3% (Table S4). To clone the hybrid male-sterility gene, the heterozygous F_1_ was successively backcrossed to the recurrent parent, T65. The pollen fertility of BC_n_F_1_ gradually increased with successive backcrosses to T65 (Table S4). In all backcrosses prior to BC_4_F_1_, T65 was continuously used as a male parent, and pollen semi-sterile BC_n_F_1_ was used as a female parent. Despite continued backcrossing, pollen fertility remained consistently low after BC_4_, whereas seed fertility of pollen semi-sterile segregants increased, and pollen semi-sterile BC_7_F_1_ plants produced fully fertile seeds. These observations suggest that the genetic factor for pollen semi-sterility does not have any deleterious effects on seed fertility. Among BC_7_F_2_ segregants derived from self-pollination of BC_7_F_1_, fully pollen-fertile plants were selected. These plants and their self-pollinated progenies were regarded as an NIL, T65*A*^*+*^*S13*^*l*^. Anthocyanin pigmentation in young T65*A*^*+*^*S13*^*l*^ seedlings confirmed the introgression of the chromosomal region around the *A* locus located on chromosome 1 in rice (for details, see Materials and Methods). The agronomic traits of the NIL and T65 were mostly similar, except for panicle number (Figure S2).

**Figure 1:**
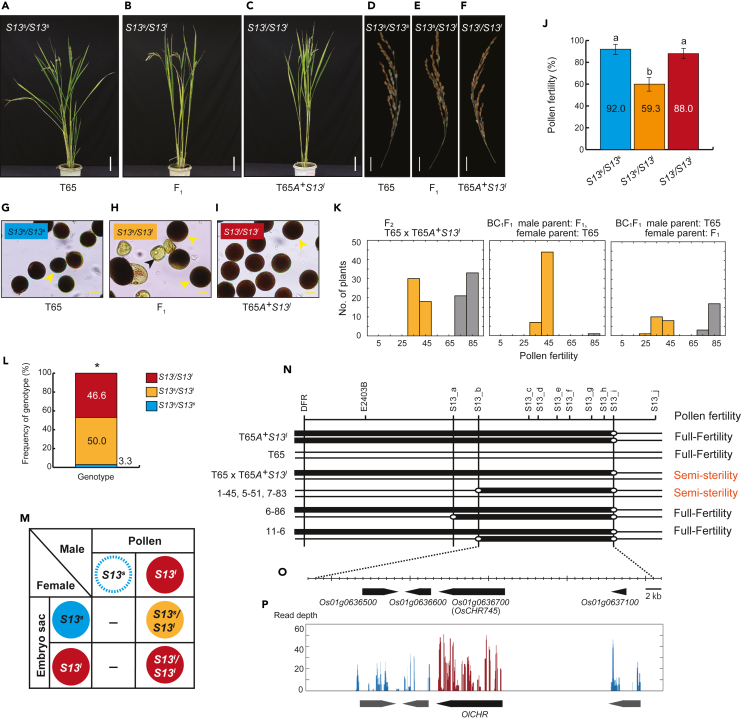
Mapping and effect of *S13* (A–C) Vegetative growth of T65*,* T65*A*^*+*^*S13*^*l*^*,* and their heterozygous F_1_. Scale bar = 10 cm. (D–F) Panicles of T65*,* T65*A*^*+*^*S13*^*l*^, and their heterozygous F_1_. Scale bar = 4 cm. (G–I) Pollen phenotypes of T65*,* T65*A*^*+*^*S13*^*l*^*,* and their heterozygous F_1_. Black and yellow arrows indicate sterile and fertile pollen grains, respectively. Scale bar = 20 μm. (J) Pollen fertility of T65*,* T65*A*^*+*^*S13*^*l*^*,* and their heterozygous F_1_. Error bar stands for SD. Values indicate pollen fertility %. Different letters show significant difference by the Tukey test at the 1% level of significance. (K) Frequency distributions of pollen fertility in F_2_ (left), BC_1_F_1_ derived from the cross of male F_1_ and female T65 plants (middle), and BC_1_F_1_ derived from the cross of male T65 and female F_1_ plants (right). Pollen semi-sterile plants are shown in yellow. (L) Allelic segregation of F_2_ derived from a heterozygous F_1_ (*S13*^*s*^*/S13*^*l*^). No. of plants with *S13*^*l*^*/S13*^*l*^*, S13*^*s*^*/S13*^*l*^ and *S13*^*s*^*/S13*^*s*^ genotypes were 14, 15 and 1, respectively. Values indicate frequency of each genotype. ∗ indicates *p* value less than 0.05 based on Fisher’s exact test expected to fit to Mendelian segregation ratio. (M) Expected pattern of gametic distribution of F_1_ and allelic composition in F_2_. The aborted allele is shown in the dotted circle. Em dash (−) indicates no tendency to produce a specific allelic combination due to abortion of *O. sativa* allele-containing pollens. (N) High-resolution mapping of the *S13* locus. Graphical genotypes of parents and recombinants are indicated by horizontal bars and lines. Bars and lines indicate chromosomes derived from W1618 (*O. longistaminata*) and T65 (*O. sativa*), respectively. Pollen fertility of each plant is shown on the right side of the graphical genotype. (O) Predicted genes in the candidate region of the *S13* locus based on the rice reference genome (Nipponbare, IRGSP1.0). (P) RNA-seq and read depth of the candidate region of the *S13* locus. RNA from a T65*A*^*+*^*S13*^*l*^ anther was used for analysis.

To confirm the effect of a gene for pollen semi-sterility, we crossed T65*A*^*+*^*S13*^*l*^ with T65 and compared F_1_ phenotypes with those of its parents. Although there were no differences in the overall plant shape and seed fertility between F_1_ and parents (T65*A*^*+*^*S13*^*l*^ and T65) (Figures 1A–1F), pollen fertility was significantly lower in F_1_ (Figure 1J). In anthers prior to flowering, there were aborted pollen grains in F_1_, while almost all pollen grains normally developed in the parents (Figures 1G–1I). In the F_2_ population derived from self-pollination of F_1_ plants, half of the plants showed pollen semi-sterility (pollen fertility, 45%–55%), and the other half showed ≥85% pollen fertility (Figure 1K). This indicated a consistent and clear-cut difference between pollen semi-sterile and fertile states; pollen semi-sterile plants and fertile plants segregated 1:1 (Figure 1K). In BC_1_F_1_ plants developed by crossing T65 as a male and an F_1_ plant as a female, pollen semi-sterile plants and fertile plants also segregated 1:1, while BC_1_F_1_ plants developed by reciprocal cross were mostly semi-sterile (Figure 1K). These results indicated that pollen semi-sterility occurs only in heterozygotes (hybrids) and that male gametes with the T65-derived allele were preferentially aborted in F_1_ heterozygotes. Thus, we named this hybrid sterility locus as *S13*, and alleles derived from T65 and W1618 as *S13*^*s*^ and *S13*^*l*^, respectively. Based on the above, the genetic action of the *S13* locus is considered a pollen-killer type causing *m*TRD.^18^ In that case, F_1_ heterozygotes are expected to produce *S13*^*l*^ homozygotes and heterozygotes (*S13*^*s*^/*S13*^*l*^) in a 1:1 ratio in the F_2_ population. We used a DNA marker, E2403B, linked with the *A* locus and confirmed that 46.6% and 50.0% of plants were homozygotes (*S13*^*l*^/*S13*^*l*^) and heterozygotes (*S13*^*s*^/*S13*^*l*^) in the F_2_ population, respectively (Figure 1L). These results indicated that the *S13*^*l*^ allele derived from W1618 is a selfish genetic element (Figure 1M) and that the *S13* locus is a hybrid sterility locus causing TRD in distantly related *Oryza* AA genome species.

### Male gametophyte development in *S13*^*s*^/*S13*^*l*^ heterozygotes

We then examined the developmental stage at which pollen abortion occurred in heterozygotes (*S13*^*s*^*/S13*^*l*^). To do so, we investigated the male gametophyte in an F_1_ hybrid of T65 and T65*A*^*+*^*S13*^*l*^ (i.e., pollen grain or microgametophyte) at three pollen developmental stages (one-nucleus, two-nuclei, and pollen mature stages, as shown in Table S5). All pollen grains normally developed at the one-nucleus stage. However, half of them showed abnormalities with nuclei degeneration at the two-nuclei stage (Table S5). The empty and shriveled pollen grains were observed at this stage (Figure S3). At the mature stage, similar to the two-nuclei stage, half of the pollen grains were empty and shriveled with I_2_-KI staining, while the other half developed normally (Figure S3). These results suggest that microspores with the *S13*^*s*^ allele were arrested after meiosis of the pollen mother cell and before mitotic cell division of the generative cell.

### High-resolution mapping of *S13*

In an F_2_ population of 102 plants, we observed clearly distinguishable pollen semi-sterile and fertile states with a 1:1 segregation pattern (Figure 1K). Therefore, we used these two states (pollen semi-sterile and fertile) for further mapping. We conducted initial linkage mapping using 300 F_2_ plants using DNA markers on the *A* (*DFR*) locus and E2403B to investigate their segregation pattern in the F_2_ population. Among all F_2_ segregants, we did not find homozygous T65-derived alleles for both loci, suggesting the elimination of T65-derived alleles at these loci (*DFR* and E2403B) in conjunction with the *S13*^*s*^ allele (Figure S4). Using the maximum likelihood method, the genetic distance between E2304B and *S13* and between *DFR* and E2403B were estimated as 0.33 cM and 0.17 cM, respectively (Figure S4).

The length of the introgressed segment from W1618 into the NIL T65*A*^*+*^*S13*^*l*^ was further examined using 10 additional DNA markers. Despite the presence of polymorphism between T65 and the original wild rice accession W1618, T65 and T65*A*^*+*^*S13*^*l*^ had the same genotype at markers *S13*_i and *S13*_j (Figure 1N). Thus, *S13*_i and *S13*_j are not present in the region introgressed from W1618 into T65. Accordingly, the *S13* candidate region can be mapped to the segment starting from E2403B and ending before *S13*_i (Figure 1N). For high-resolution mapping, we used another segregating population of 2011 plants derived from the cross between T65 and T65*A*^*+*^*S13*^*l*^. Then, a total of five recombinants (1–45, 5–51, 6–86, 7–83, and 11–6) between E2403B and *S13*_e were identified (Figure 1N). Among these recombinants, three (1–45, 5–51, and 7–83), which were heterozygous for the region between markers *S13*_c and *S13*_h, showed pollen semi-sterility, whereas the other two (6–86 and 11–6), which were homozygous in that region, exhibited pollen fertile phenotype. These results suggest that the *S13* locus, which can cause pollen semi-sterility and *m*TRD, can be delimited to a 40 kb region between *S13*_b and *S13*_i. This region was overlapping to the *qHMS1* locus causing hybrid sterility between *O. sativa* and another distantly related species, *O. meridionalis*.^29^

### Candidate genes in *S13*

In this region, there are four open-reading frames in the reference genome of rice (Nipponbare, IRGSP1.0): *Os01g0636500* (Polygalacturonase PG2), *Os01g0636600* (hypothetical conserved gene), *Os01g0636700* (Chromatin remodeling factor 745), and *Os01g0637100* (F-box protein 29) (Figure 1O). Among them, positions of *Os01g0636700* and *Os01g0637100* were identical to those of *hpt* and *hpa* genes in the *qHMS1* locus, respectively.^29^ NGS revealed that T65 had the same sequence as the reference genome in this region (sequence data were deposited in the public database (https://www.ddbj.nig.ac.jp), under the BioProject, PRJDB17754). We then compared the genomic sequences of T65*A*^*+*^*S13*^*l*^ to the reference. Additionally, we used RNA-seq of T65*A*^*+*^*S13*^*l*^ to identify transcriptomic structures in the corresponding region (Figure 1P). Despite the existence of various polymorphisms (SNPs and InDels) within the delimited 40 kb region, variants were mostly located in noncoding regions. A conspicuous difference between Nipponbare and T65*A*^*+*^*S13*^*l*^ was the presence of one SNP in *Os01g0636700*, which results in a premature stop codon in Nipponbare but not in T65*A*^*+*^*S13*^*l*^ (Figure S5). In addition to the SNP, a deletion of approximately 1.3 kb in the homologous gene of *Os01g0637100* of T65*A*^*+*^*S13*^*l*^ was identified. Although You et al.^29^ just recently reported that the approximate 1.3 kb insertion causes disfunction of the *HPA* gene in the *qHMS1* locus, we could not determine the effect of this deletion/insertion on the function of genes in this study. Taken together, among the four genes, we considered *Os01g0636700* for the first target gene to be analyzed, because the SNP on this gene may cause the functional change between T65 and T65*A*^*+*^*S13*^*l*^. In the RAP Database, *Os01g0636700* is named chromatin remodeling factor 745 (*OsCHR745,* https://rapdb.dna.affrc.go.jp); accordingly, we designated its homologue in *O. longistaminata* as *OlCHR* (Figure 1P), although *OlCHR* might be identical to *HPT* gene according to its gene position. The comparison of amino acid sequences between *OsCHR745* and *OlCHR* revealed that the earlier stop codon in *OsCHR745* results in the deletion of the helicase domain, truncating into 470 aa from 902 aa. (Figure S6).

The expression of *OlCHR* and *OsCHR745* in anthers before flowering was confirmed using RT-PCR (Figure S7). The public database Rice Xpro (https://ricexpro.dna.affrc.go.jp/) showed that *OsCHR745* is expressed in various tissues including young anthers (Figure S7). Rahman et al. (2019) demonstrated the expression of *OsCHR745* in sperm cells (Figure S7).

### CRISPR-Cas9-mediated mutagenesis restored hybrid male fertility

**Figure 2:**
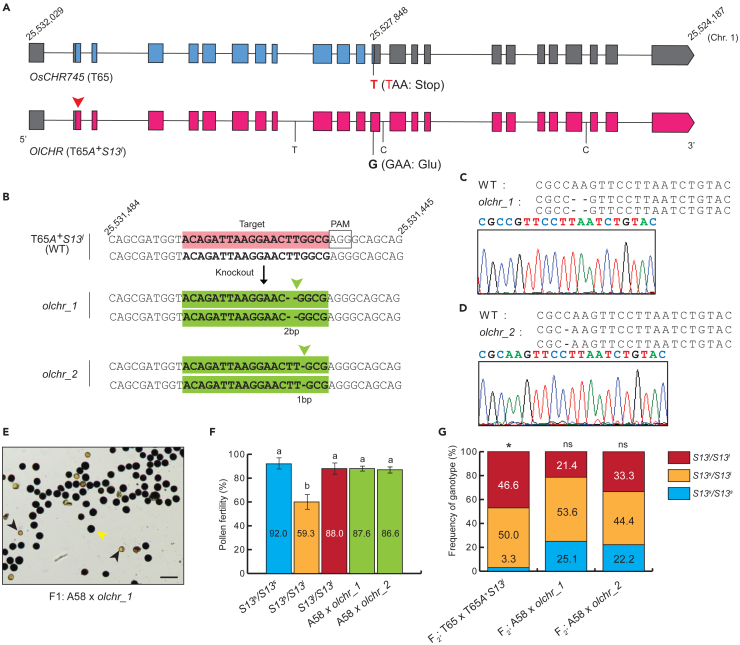
Gene identification of the *S13* locus (A) Gene models of *OsCHR745* and *OlCHR* in *O. sativa* and *O. longistaminata*, respectively. The structure of the candidate gene in *O. sativa* is truncated due to the presence of an earlier stop codon (TTA), with adenine at the position 25,527,848 replaced by cytosine in the *O. longistaminata* allele. Red arrowhead pointing downward indicates the target region for CRISPR/Cas9-induced mutation. (B) Genotypes of target sites and flanking regions in T65*A*^*+*^*S13*^*l*^ and mutants (*olchr*_1 and *olchr*_2). Pink box indicates the 20-bp-long target sites beside the PAM sequence (AGG), which guides the Cas9 enzyme. Green boxes indicate the corresponding target sites after mutation induction. (C and D) Sanger sequences of mutants. (E) Pollen phenotypes of F_1_ (A58 x *olchr*_1). Black and yellow arrowheads represent sterile and fertile pollens, respectively. Scale bar = 100 μm. (F) Pollen fertility of heterozygous F_1_ (A58 x *olchr* mutants). The error bar represents SD. Values indicate pollen fertility %. Different letters show significant difference by the Tukey test at the 1% level of significance. (G) Allelic segregation of F_2_ derived from heterozygous F_1_ (A58 x *olchr* mutants). Values indicate frequency of each genotype. 30, 27, and 9 plants were used for F_2_ populations derived from T65 x T65*A*^*+*^*S13*^*l*^, A58 x *olchr*_1, and A58 x *olchr*_2, respectively. ∗ indicates *p* value less than 0.05 based on Fisher’s exact test expected to fit to Mendelian segregation ratio.

We hypothesized that the premature stop codon in *OsCHR745* produces a truncated and nonfunctional protein in T65, while *OlCHR* produces the functional protein; this change would result in different functions of the *S13*^*s*^ and *S13*^*l*^ alleles (Figure 2A). To confirm this hypothesis, we developed CRISPR-Cas9 mutants of *OlCHR* in T65*A*^*+*^*S13*^*l*^. The guide RNA was designed to recognize a specific target on the 1^st^ exon of *OlCHR* in T65*A*^*+*^*S13*^*l*^. Then, we produced mutants with 2- and 1-bp deletions upstream of the PAM sequence: *olchr*_1 and *olchr*_2, respectively (Figures 2B–2D). These mutants were crossed with A58 (a variety of *O. sativa*). A58 did not show hybrid pollen sterility and TRD in the *A* locus tightly linked with the *S13* locus in the F_2_ population when it was crossed with T65, while it showed hybrid pollen sterility and TRD when it was crossed with T65*S13*^*l*^ (Figure S8). This result indicated that A58 has the *S13*^*s*^ allele in the *S13* locus. We also confirmed that there are no SNPs and InDels in the coding region of the candidate genes between T65 and A58, thus we used this variety as the representative of the *S13*^*s*^ carrier. The pollen fertility of F_1_ hybrids between A58 and *olchr*_1 and between A58 and *olchr*_2 was 87.6% and 86.6%, respectively (Figures 2E and 2F). These values were not significantly different from the pollen fertility of T65 and T65*A*^*+*^*S13*^*l*^ (Figure 2F), indicating that hybrid male sterility did not occur in these cross combinations. Thus, *OlCHR* may be related to *S13* locus-mediated hybrid sterility. In the *S13* locus-mediated hybrid sterility system, there is preferential transmission of the *S13*^*l*^ allele via male gamete abortion in hybrids (*S13*^*s*^/*S13*^*l*^). Thus, we examined the segregation pattern of F_2_ populations derived from F_1_ plants to confirm if there was preferential transmission of the *S13*^*l*^ allele. In the F_2_ populations, the segregation patterns were not significantly different from the Mendelian 1:2:1 ratio (Figure 2G), indicating that *m*TRD did not occur in F_2_ populations derived from the cross between T65*A*^*+*^*S13*^*l*^ and mutants (*olchr*_1 and *olchr*_2). These results strongly suggested that *OlCHR* is the causative gene for *S13* locus-mediated hybrid male sterility.

### Widespread stop codon allele

**Figure 3:**
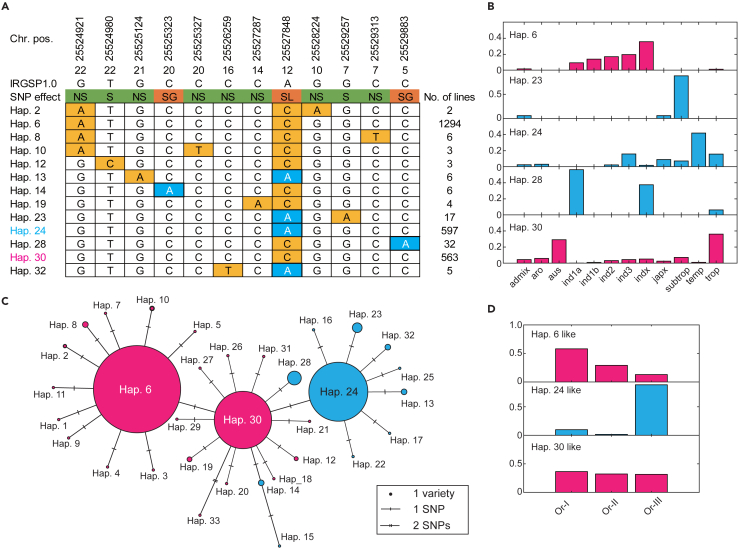
Haplotype analysis of *OlCHR* (A) Haplotypes classified by SNPs in the coding region of *OlCHR* using rice 3k data. Only haplotypes with >2 varieties are shown. Singleton SNPs are not shown. Number of exons where SNPs were located, reference allele, and expected SNP effect are shown below the chromosomal position. NS, S, SG, and SL indicates non-synonymous, synonymous, stop codon gained, and stop codon lost mutations, respectively. In the SNP table, nonreference type variants are shown in orange. Variants causing the premature stop codon are shown in blue. Haplotypes whose names are shown in blue and magenta (Hap. 24 and Hap. 30, respectively) indicates haplotypes identical to *OsCHR745* and *OlCHR* sequences. (B) Frequency of subpopulations in haplotypes 6, 23, 24, 28, and 30. Haplotypes with and without the premature stop codon are shown in blue and magenta, respectively. The subpopulation classification was based on Rice SNP-Seek database (https://snp-seek.irri.org/). Y axis indicates proportion of each subpopulation in the haplotype. (C) Haplotype network. The relative number of varieties included in each haplotype is indicated by the area of each circle. The length of the line connecting two circles represents the number of SNPs between two haplotypes. (D) Haplotype analysis of *O. rufipogon* classified by SNPs in the *OlCHR* homologue based on the Oryza Genome database (http://viewer.shigen.info/oryzagenome21detail/index.xhtml). Haplotypes with and without stop codon are shown in magenta and blue, respectively.

In the above analysis, we identified that *OlCHR* and a premature stop codon in its homologue *OsCHR745* causes functional changes in alleles of the *S13* locus. *OlCHR* mutagenesis resulted in two mutants, *olchr*_1 and *olchr*_2, which did not induce male sterility in hybrids crossed with the *S13*^*s*^ possessing variety, A58. If the *S13* locus-mediated hybrid sterility is explained by Killer–Protector system or Killer–Responder system, these results suggested that the premature stop codon in *OsCHR745* induces loss of functional change in the Killer action. To infer allele divergence in *OsCHR745*, we first used public databases (Rice SNP-Seek and Oryza Genome) to examine the presence of the premature stop codon in the Asian rice gene pool (*O. sativa* and *O. rufipogon*). Among 3,024 *O. sativa* varieties, 2,558 had available SNP data for *OsCHR745* in the database. These varieties were further classified by SNPs in CDS in *OsCHR745* and a total of 33 haplotypes were identified (Figure S9). Among them, five (Hap.6, Hap.23, Hap.24, Hap.28, and Hap.30) consisted of >10 varieties (Figures 3A and S9). For 20 haplotypes, only one variety belongs to the haplotype (Figure S9). Among all haplotypes, eight possess the premature stop codon (TAA), having a Thymine at 25,527,848 bp on chromosome 1. The frequency of varieties with this stop codon corresponded to nearly one-fourth of the *O. sativa* population. Two other SNPs that also cause a premature stop codon in the 5^th^ and 20^th^ exons of *OsCHR745* were newly found in Hap. 28 and Hap. 14, respectively, suggesting that *OsCHR745* was also nonfunctional in these haplotypes (Figure 3A). To examine the relationship between haplotypes and subpopulations in *O. sativa*, we classified varieties in each haplotype based on subpopulations to which they belong (Figures 3B and 3C, the name of each subpopulation is based on the Rice SNP-Seek Database). This analysis revealed subpopulation-specific haplotype distribution. For Hap. 23 and 24, *subtrop* and *temp* varieties are widespread, respectively (Figure 3B). For Hap. 28, most varieties belong to *ind1a* and *indx* subpopulations. For Hap. 6, which does not have a premature stop codon, varieties mostly belonged to indica subpopulations (*ind1a*, *ind1b*, *ind2*, *ind3*, and *indx*). For Hap. 30, varieties belonged to *aus* and *trop* subpopulations. In summary, the premature stop codon is commonly observed in *subtrop* and *temp* subpopulations but at low frequency in other subpopulations. The presence of SNPs were confirmed by PCR and sequencing using World Rice Core Collection (WRC) and Japan Rice Core Collection (JRC) provided by NARO (National Agriculture and Food Research Organization), Japan (Figures S10 and S11). Additionally, we classified *O. rufipogon* based on available haplotype information of two SNPs (25,524,921 and 25,527,848) which are overlapping with SNPs from *O. sativa*. Among a total of 446 *O. rufipogon* accessions, 431 were used because of availability of the SNP dataset in the public database. Based on the two SNPs, three haplotypes (A-C, G-A, and G-C on 25,524,921 and 25,527,848) were found. These three haplotypes were named as Hap. 6-like, Hap. 24-like, and Hap. 30-like, based on the similarity to haplotypes in *O. sativa*. Hap. 6-like haplotypes were observed in all three *O. rufipogon* supspecies (Or-I, Or-II, and Or-III), though frequency of varieties of Or-I were high. For Hap. 23-like, Or-III varieties were widespread. For Hap. 30-like, varieties from three subpopulations were found (Figure 3D). These results suggest that Hap. 30 (Hap. 30-like) is the ancestral state of *OsCHR745* and that the premature stop codon evolved before domestication of *O. sativa*.

### Identification of *S13*^*n*^ neutral allele

**Figure 4:**
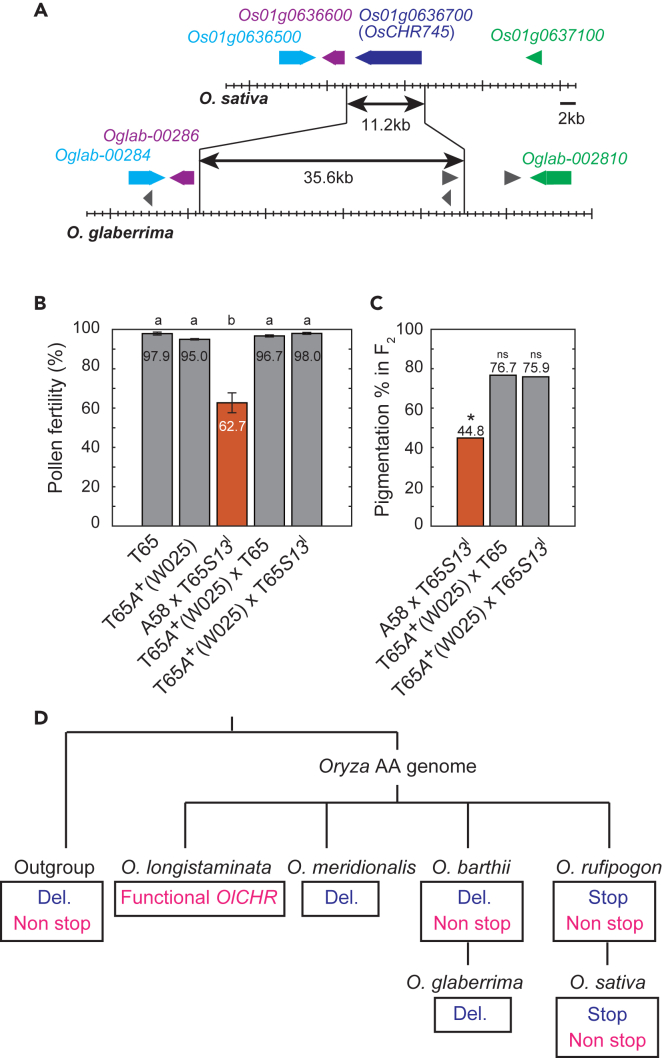
Analysis of neutral alleles (*S13*^*n*^) in W025 (*O. glaberrima*) (A) Chromosomal region of the deletion and insertion detected in *O. glaberrima* genome in the candidate region for the *S13* locus based on a public database (Rice Genome Hub, https://rice-genome-hub.southgreen.fr/). The deletion and insertion are shown by bars with arrowheads below the gene models predicted in the rice reference genome (IRGSP1.0). Each pair of homologous genes follows the same color code. Genes without homologous pairs are indicated by gray arrowheads. (B) Pollen fertility. The error bar represents SD. Different letters show significant difference by the Tukey test at the 1% level of significance. (C) Frequency of pigmented plants in the F_2_ population. Seedling pigmentation in the F_2_ population was used for checking distortion of the *A* (*Anthocyanin activator*) locus linked with the *S13* locus. Orange bars indicate semi-sterile pollen and segregation ratio distorted cross combination. Error bar stands for SD. (D) Evolutionary relationship of species in the genus *Oryza* with putative alleles of the *OlCHR* homologue. The tree topology is based on Wang et al. (2023b). For outgroup species, *O. minuta*, *O. rhizomatis*, *O. alta* (or *O. latifolia*), *O. punctata*, *O. grandiglumis*, *O. australiensis*, and *O. brachyantha* were used (for detail see, Table S1).

To clarify the evolution of the *S13* locus, we analyzed the distribution of *OsCHR745* homologues within *Oryza* AA genome species and outside of this species group. Interestingly, no DNA fragment was amplified through PCR using primers designed to anneal to the surrounding regions of the premature stop codon in W025 (*O. glaberrima*, African cultivated rice). Next, we analyzed the genome structure of *O. glaberrima* using the public database Rice Genome Hub (https://rice-genome-hub.southgreen.fr/) and found that an 11.2 kb chromosomal region containing *OsCHR745* was deleted, and a 35.6 kb chromosome fragment, which does not contain the *OsCHR745* homologue, was present (Figure 4A). This suggests the lack of a homologue of *OsCHR745* in *O. glaberrima*. To confirm this finding, we sequenced the W025 genome using NGS. No short reads were mapped to the chromosomal region corresponding to *OsCHR745,* corroborating our previous result. This prompted us to examine the effect of the insertion/deletion on *S13* locus-mediated hybrid male sterility. We developed an NIL of W025 using W025 as a donor parent and T65 as a recurrent parent through six successive backcrosses. The developed NIL T65*A*^+^(W025) was crossed with T65 or T65*S13*^*l*^ (an NIL with a nonfunctional gene *A* and the allele *S13*^*l*^; for details, please see Materials and Methods) to examine the allelic status of the *S13* locus in T65*A*^+^(W025). In these test crosses, both F_1_ plants showed >90% pollen fertility. Additionally, the segregation ratio of anthocyanin pigmentation caused by the *A*^+^ gene was not distorted, showing a Mendelian ratio (3:1, Figures 4B and 4C). These results indicate that T65*A*^+^(W025) has an additional allele, different from both *S13*^*s*^ and *S13*^*l*^ alleles, recognized as the neutral allele *S13*^*n*^ (*n* for neutral^48^^,^^49^), which causes no allelic preferential abortion in heterozygosity (*S13*^*s*^/*S13*^*n*^ and *S13*^*l*^/*S13*^*n*^). If the *S13* locus-mediated hybrid sterility is explained by a Killer–Protector system or Killer–Receptor system, the lack of the *OsCHR745* homologue in W025 induced a loss of functional change in the Killer action, similar to the case of CRISPR-Cas9-mediated mutagenesis.

We then examined the presence of *OlCHR* homologues in *Oryza* species via PCR and sequencing. Figure 4D shows the distribution of *OlCHR* homologues in *Oryza* species. In the African rice gene pool (*O. glaberrima* and *O. barthii*), only a few *O. barthii* accessions had the *OlCHR* homologue. Interestingly, in *O. meridionalis*, which is shown to have the *HPT* gene in You et al.^29^ the *OlCHR* homologue was not detected in any accessions examined. To confirm this result, we analyzed the genome structure of *O. meridionalis* using the public database Rice Genome Hub (https://rice-genome-hub.southgreen.fr/). In the public database, a large deletion (about 58.5 kb) which causes the lack of homologues of *OlCHR* (*HPT*) and of *Os01g0637100* (*HPA*) was observed (Figure S12). This deletion was also confirmed in all 18 accessions of *O. meridionalis* in our NGS analysis and following the PCR amplification (Table S1). In outgroup species, an *OlCHR* homologue was found in some varieties of different species. These results suggest that the *OlCHR* homologue originated outside of *Oryza* AA genome species and that this gene was deleted in some species. In particular, in African cultivated rice (*O. glaberrima*) and in an Australian wild rice species (*O. meridionalis*), the lack of *OlCHR* or its homologue *HPT* seemed to be widely distributed.

## Discussion

In rice, most hybrid sterility loci in intraspecific hybrids of *O. sativa* conform to the one-locus sporogametophytic interaction model, in which gametes with one of the two alleles preferentially transmit due to the abortion of gametes with alternative alleles in heterozygotes.^16^^,^^17^^,^^18^^,^^19^^,^^20^^,^^21^^,^^22^^,^^23^^,^^24^^,^^25^^,^^26^^,^^27^^,^^28^^,^^29^^,^^30^^,^^31^^,^^32^^,^^33^^,^^34^^,^^35^^,^^36^^,^^37^^,^^38^^,^^39^^,^^40^^,^^41^^,^^42^^,^^43^^,^^44^^,^^45^^,^^46^^,^^47^^,^^48^^,^^49^^,^^50^ Genes following this one-locus sporogametophytic interaction model cause TRD in the next generation, and they are considered selfish elements in the population. The molecular mechanism of allele-specific gamete abortion can generally be explained by two possible genetic models: Killer–Protector and Killer–Responder.^12^^,^^32^ Although extensive studies have identified the involvement of one of these two models in intraspecific hybrid sterility in Asian cultivated rice, no causative genes for hybrid sterility and TRD in distantly related *Oryza* AA genome species, such as a combination between *O. sativa* and *O. longistaminata* have been cloned so far. In the present study, we successfully cloned the causative gene for the hybrid sterility locus *S13*, which induces pollen semi-sterility and male-specific TRD (*m*TRD) in hybrids between *O. sativa* and its distantly related relative, *O. longistaminata*. Furthermore, we discovered that *OlCHR* is the causative gene for the *S13* locus-mediated hybrid sterility (Figures 1N–1P). The position *OlCHR* is identical to that of *HPT* gene which is recently shown to act as the “toxin” in the toxin-antidote system of hybrid sterility in the cross between *O. sativa* and *O. meridionalis*.^29^ A naturally occurring lack of the *OlCHR* in *O. glaberrima* produces the allele (*S13*^*n*^), which does not exhibit sterility or *m*TRD in F_1_ plants developed by crossing with either an *S13*^*s*^ or *S13*^*l*^ carrier (Figures 4B and 4C). These results strongly indicate that *OlCHR* functions as a Killer gene. Considering Killer–Protector or Killer–Receptor systems, *S13*^*n*^ allele lacks the Killer function, while it still has a Protector or it lacks a Receptor, and this provides compatibility between *S13*^*n*^ and *S13*^*l*^ carriers (Figures 4B and 4C); the remaining Killer action from the *S13*^*l*^ carrier is diminished in the presence of the functional Protector of the *S13*^*n*^ carrier, or in the absence of active Receptor in the *S13*^*n*^ allele in the *S13* locus. It is inconceivable that *OlCHR* had a Protector function, because the *S13*^*n*^ carrier, T65*A*^+^(W025) did not exhibit sterility in hybrids with T65*A*^*+*^*S13*^*l*^; if *OlCHR* had a Protector function, gametes with the allele from T65*A*^+^(W025) would not be protected in this cross combination. Therefore, this study confirms the presence of Killer system in hybrid sterility found in *O. longistaminata*, a distantly related *Oryza* AA genome species.

Although we identified the Killer gene in this study, the identification of the Protector or Responder remains pending in our study. Currently, two different genetic models, Killer–Protector and Killer–Responder, are possible explanations for *S13* locus-mediated hybrid sterility. Although we cannot definitively determine which model is correct, it is likely that either a Protector or Responder gene exists in the *S13* region. As such, the other three genes (*Os01g0636500*, *Os01g0636600*, and *Os01g0637100*), or their homologues in *O. longistaminata*, are potential candidates for the Protector or Responder functions. Among them, *Os01g0636500* and *Os01g0636600* do not exhibit polymorphisms in the coding sequences between T65*A* and T65*A*^*+*^*S13*^*l*^. In contrast, a deletion of approximately 1,300 bp in the homologous gene of *Os01g0637100* of T65*A*^*+*^*S13*^*l*^ was identified. While the gene expression level is low, this gene is also expressed in various tissue (Figure S13). Recently, You et al.^29^ showed that *HPA* (a homologue of *Os01g0637100*) shows the “antidote” function in the toxin-antidote hybrid sterility system of *qHMS1* found between *O. sativa* and *O. meridionalis*. This report strongly showed that the homologous gene of *Os01g0637100* of T65*A*^*+*^*S13*^*l*^ is the Protector, though it is necessary to confirm the effect of this gene in *S13* locus-mediated hybrid sterility.

*OlCHR* is a homologue of *OsCHR745*, a Snf2 family protein found in *O. sativa*. The highly diverse ATP-dependent Snf2 family proteins are involved in various chromatin remodeling processes, including transcription regulation, replication, DNA repair, DNA recombination, and chromatin unwinding (https://www.ncbi.nlm.nih.gov/Structure/cdd/PF00176, Guo et al.^51^; Hu et al.^52^). Many Snf2 family genes, including *OsCHR745*, exhibit tissue-specific expression patterns and are relatively highly expressed in young panicles and callus.^52^ Guo et al.^51^ also demonstrated that *OsCHR745* is highly expressed in the panicle (P2 stage; 3–5 cm) and mature ovary. These expression patterns are consistent with the observed role of this gene in pollen abortion. In fact, a relationship between chromatin state and male gametogenesis has been suggested. Pinon et al.^53^ reported that SET DOMAIN GROUP2 (SDG2)-mediated H3K4me3 deposition is required for post-meiotic microspore chromatin landscape, the second mitotic cell cycle progression, and the activation of transposable elements (LTR retrotransposon *ATLANTYS1*) during *Arabidopsis* male gametogenesis. Moreover, a loss-of-function *sdg2-1* mutant affects pollen development, such as pollen germination and pollen tube elongation. Phylogenetic analysis of Snf2 family proteins from *O. sativa*, *Arabidopsis thaliana*, and *Sorghum bicolor* revealed that *OsCHR745* belongs to a common group called SSO1653-like, along with *CHR24* from *Arabidopsis thaliana*. It is closely located in the same ERCC6 subfamily.^52^^,^^54^ According to Shaked et al.^55^ and Knizewski et al.,^56^ the *Arabidopsis* Snf2/ERCC6 subfamily proteins AT2G18760 (*CHR8*) and AT5G63950 (*CHR24*) are involved in responding to DNA damage, which can frequently occur under normal conditions and various stress conditions, such as ultraviolet light, heat, drought, and ROS-induced stress, etc.^57^ These findings suggest the involvement of DNA damage repair in male gametogenesis. However, further studies are necessary to reveal how chromatin remodeling are related to the phenotypic deficiencies observed during male gametogenesis in hybrids.

In the present study, we identified an *OlCHR* homologue in *Oryza* outgroup species (Figure 4D). This suggests that *OlCHR* homologues evolved before the divergence of *Oryza* AA genome species. In certain *Oryza* AA genome species, including African cultivated rice (*O. glaberrima*), the chromosomal region corresponding to *OlCHR* was deleted. Furthermore, we discovered that a variety of *O. glaberrima* possesses a neutral allele (*S13*^*n*^) in the *S13* locus. Based on these results, we can speculate that the lack of the *OlCHR* homologue, causing the allelic change from *S13*^*l*^ to *S13*^*n*^, occurred and became fixed in African rice species. Interestingly, in *O. meridionalis*, we identified the 58.5 kb deletion which causes the lack of homologues of *OlCHR* (*HPT*) and *Os01g0637100* (*HPA*)*.* Considering that *HPT* and *HPA* functioned as the toxin and the antidote in hybrid sterility locus *qHMS1*, respectively,^29^ the lack of these two genes may produce the “killed” allele. The male gametes with this allele are preferentially aborted in heterozygous of *qHMS1* (*S13*). Therefore, our analysis suggested that *qHMS1* is polymorphic in *O. meridionalis* and, if so, *qHMS1* may have shown its function within *O. meridionalis*, though more detailed analysis is necessary to confirm this. Juliano et al.^58^ showed that some within-species hybrids of *O. meridionalis* shows pollen sterility. This may indicate that some hybrid sterility genes are polymorphic in *O. meridionalis*. Our haplotype analysis also provides a clue to understand the evolutionary process of the emergence of the premature stop codon in *OsCHR745* and subsequent distribution of this truncated gene. In *O. rufipogon*, a Hap. 30-like variant without an SNP causing a premature stop codon appears to be ancestral. In the Or-III subpopulation of *O. rufipogon*, a variety with a premature stop codon was frequently detected. This suggests that the premature stop codon in *OsCHR745* evolved in *O. rufipogon* and subsequently spread to *O. sativa* through the domestication process. Huang et al.^59^ proposed that *O. sativa* indica and japonica descend from Or-I and Or-III, respectively. Consequently, the different SNP frequencies in *OsCHR745* between *O. sativa* subpopulations may reflect their domestication history.

In hybridization breeding, reproductive isolation prevents effective gene exchange between species. In some distantly related rice species, including *O. longistaminata*, useful genes responsible for abiotic and biotic stresses have been described (for *O. longistaminata*, see Tong et al.^39^; Khush et al.^60^; Atwell et al.^61^); accordingly, these species are considered valuable genetic resources for improving *O. sativa*. In the present study, we conducted CRISPR-Cas9 mutagenesis and developed a nonfunctional Killer allele in the hybrid sterility locus, which did not induce *S13* locus-mediated hybrid sterility. Previous studies by Xie et al.,^23^ Koide et al.,^24^ You et al.,^29^ Zhou et al.,^37^ and Xie et al.^62^ effectively generated neutral alleles in hybrid sterility loci using CRISPR-Cas9 or heavy ion beam mutagenesis. Despite the numerous hybrid sterility genes present in *Oryza*, a mutagenesis-based approach may overcome species barriers and facilitate the transfer of beneficial traits from *Oryza* wild relatives.

### Limitations of the study

In this study, we identified *OlCHR*, a gene with a Killer function in a hybrid sterility system between two rice species. However, to fully characterize this system, future studies should aim to identify the Protector or Responder gene. Additionally, the molecular function of *OlCHR* remains unclear. As *OlCHR* shares homology with a gene encoding a chromatin remodeling factor, future studies should explore the relationship between chromatin status and hybrid male sterility.

## STAR★Methods

### Key resources table

**Table:** 

REAGENT or RESOURCE	SOURCE	IDENTIFIER
**Bacterial and virus strains**		

DH5α competent cell	TaKaRa	Cat#9057
*Agrobacterium tumefaciens* LBA4404	TaKaRa	Cat#9115

**Biological samples**		

*Oryza australiensis* W0008	National Bioresource Project, MEXT, Japan	IRGC100882
Oryza grandiglumis W0613	National Bioresource Project, MEXT, Japan	W0613
Oryza punctata W1024	National Bioresource Project, MEXT, Japan	W1024
Oryza alta or Oryza latiforia W1182	National Bioresource Project, MEXT, Japan	IRGC105143
Oryza grandiglumis W1194	National Bioresource Project, MEXT, Japan	IRGC105144
Oryza minuta W1213	National Bioresource Project, MEXT, Japan	IRGC100174
Oryza longiglumis W1220	National Bioresource Project, MEXT, Japan	W1220
Oryza minuta W1331	National Bioresource Project, MEXT, Japan	IRGC101132
Oryza brachyantha W1401	National Bioresource Project, MEXT, Japan	IRGC105150
Oryza australiensis W1628	National Bioresource Project, MEXT, Japan	W1628
Oryza brachyantha W1711	National Bioresource Project, MEXT, Japan	IRGC104156
Oryza rhizomatis W1805	National Bioresource Project, MEXT, Japan	W1805
Oryza longistaminata W1618	National Bioresource Project, MEXT, Japan	W1618
Oryza meridionalis W1298	National Bioresource Project, MEXT, Japan	W1298
Oryza meridionalis W1561	National Bioresource Project, MEXT, Japan	IRGC105605
Oryza meridionalis W1627	National Bioresource Project, MEXT, Japan	IRGC104087
Oryza meridionalis W1629	National Bioresource Project, MEXT, Japan	IRGC104088
Oryza meridionalis W1631	National Bioresource Project, MEXT, Japan	IRGC104089
Oryza meridionalis W1635	National Bioresource Project, MEXT, Japan	IRGC104092
Oryza meridionalis W2069	National Bioresource Project, MEXT, Japan	W2069
Oryza meridionalis W2080	National Bioresource Project, MEXT, Japan	W2080
Oryza meridionalis W2081	National Bioresource Project, MEXT, Japan	W2081
Oryza meridionalis W2103	National Bioresource Project, MEXT, Japan	W2103
Oryza meridionalis W2116	National Bioresource Project, MEXT, Japan	W2116
Oryza barthii W0651	National Bioresource Project, MEXT, Japan	W0651
Oryza barthii W0652	National Bioresource Project, MEXT, Japan	W0652
Oryza barthii W0659	National Bioresource Project, MEXT, Japan	W0659
Oryza barthii W0691	National Bioresource Project, MEXT, Japan	W0691
Oryza barthii W0828	National Bioresource Project, MEXT, Japan	W0828
Oryza barthii W1588	National Bioresource Project, MEXT, Japan	W1588
Oryza barthii W0653	National Bioresource Project, MEXT, Japan	W0653
Oryza barthii W0679	National Bioresource Project, MEXT, Japan	W0679
Oryza barthii W0687	National Bioresource Project, MEXT, Japan	W0687
Oryza barthii W0692	National Bioresource Project, MEXT, Japan	W0692
Oryza barthii W0694	National Bioresource Project, MEXT, Japan	W0694
Oryza barthii W0827	National Bioresource Project, MEXT, Japan	W0827
Oryza barthii W0830	National Bioresource Project, MEXT, Japan	W0830
Oryza barthii W0831	National Bioresource Project, MEXT, Japan	W0831
Oryza barthii W0832	National Bioresource Project, MEXT, Japan	W0832
Oryza barthii W0833	National Bioresource Project, MEXT, Japan	W0833
Oryza barthii W0834	National Bioresource Project, MEXT, Japan	W0834
Oryza barthii W0835	National Bioresource Project, MEXT, Japan	W0835
Oryza barthii W1407	National Bioresource Project, MEXT, Japan	W1407
Oryza barthii W1411	National Bioresource Project, MEXT, Japan	W1411
Oryza barthii W1430	National Bioresource Project, MEXT, Japan	W1430
Oryza barthii W1434	National Bioresource Project, MEXT, Japan	W1434
Oryza barthii W1454	National Bioresource Project, MEXT, Japan	W1454
Oryza barthii W1574	National Bioresource Project, MEXT, Japan	W1574
Oryza barthii W1605	National Bioresource Project, MEXT, Japan	W1605
Oryza barthii W1607	National Bioresource Project, MEXT, Japan	W1607
Oryza barthii W1611	National Bioresource Project, MEXT, Japan	W1611
Oryza barthii W1613	National Bioresource Project, MEXT, Japan	W1613
Oryza barthii W1616	National Bioresource Project, MEXT, Japan	W1616
Oryza barthii W1646	National Bioresource Project, MEXT, Japan	W1646
Oryza barthii W1648	National Bioresource Project, MEXT, Japan	W1648
Oryza barthii W1702	National Bioresource Project, MEXT, Japan	W1702
Oryza barthii W1704	National Bioresource Project, MEXT, Japan	W1704
Oryza barthii W1709	National Bioresource Project, MEXT, Japan	W1709

**Chemicals, peptides, and recombinant proteins**		

DNeasy Plant Mini Kit	Qiagen	Cat#69104
RNeasy Plant Mini Kit	Qiagen	Cat#74904
tris(hydroxymethyl)aminomethane	nacalai tesque	Cat#35406-91
EDTA	nacalai tesque	Cat#151-05
Potassium Chloride	WAKO	Cat#163-03545
Agarose	Nippon Gene	Cat#318-01195
100bp DNA LadderPLUS	Nippon Genetics	Cat#MWD100P
LB-BROTH	Shioyae-muesu	Cat#391-00871
Chu(N6) Medium Salt Mixture	Shioyae-muesu	Cat#391-02021
Murashige and Skoog Plant Salt Mixture	Shioyae-muesu	Cat#392-00591
Gelrite	WAKO	Cat#075-05655

**Deposited data**		

Genome sequence of Oryza sativa: T65A+S13l	This study	PRJDB17754 (https://www.ddbj.nig.ac.jp/index.html)
Genome sequence of *Oryza meridianalis*: W1297	DNA Data Bank of Japan	DRR058012
Genome sequence of *Oryza meridianalis*: W1300	DNA Data Bank of Japan	DRR058013
Genome sequence of *Oryza meridianalis*: W1625	DNA Data Bank of Japan	DRR058014
Genome sequence of *Oryza meridianalis*: W1627	DNA Data Bank of Japan	DRR058016
Genome sequence of *Oryza meridianalis*: W1631	DNA Data Bank of Japan	DRR058017
Genome sequence of *Oryza meridianalis*: W1635	DNA Data Bank of Japan	DRR058018
Genome sequence of *Oryza meridianalis*: W1638	DNA Data Bank of Japan	DRR058019
Genome sequence of *Oryza meridianalis*: W2069	DNA Data Bank of Japan	DRR058020
Genome sequence of *Oryza meridianalis*: W2071	DNA Data Bank of Japan	DRR058021
Genome sequence of *Oryza meridianalis*: W2077	DNA Data Bank of Japan	DRR226067
Genome sequence of *Oryza meridianalis*: W2079	DNA Data Bank of Japan	DRR058022
Genome sequence of *Oryza meridianalis*: W2080	DNA Data Bank of Japan	DRR058023
Genome sequence of *Oryza meridianalis*: W2081	DNA Data Bank of Japan	DRR058024
Genome sequence of *Oryza meridianalis*: W2100	DNA Data Bank of Japan	DRR226069
Genome sequence of *Oryza meridianalis*: W2103	DNA Data Bank of Japan	DRR058025
Genome sequence of *Oryza meridianalis*: W2105	DNA Data Bank of Japan	DRR058026
Genome sequence of *Oryza meridianalis*: W2112	DNA Data Bank of Japan	DRR058027
Genome sequence of *Oryza meridianalis*: W2116	DNA Data Bank of Japan	DRR058029

**Experimental models: Organisms/strains**		

Oryza sativa: T65A+S13l	This study	N/A
Oryza sativa: T65A+(W025)	This study	N/A
Oryza sativa: T65S13l	This study	N/A
Oryza sativa: T65wx	Kinoshita 1995	N/A
Oryza sativa: A58	Plant Breeding lab., Hokkaido University, Japan	N/A

**Oligonucleotides**		

DFR Forward: aggtgcacgtagctcaaaccta	This paper	N/A
DFR Reverse: agctaccttgcacttggtgatg	This paper	N/A
E2403B Forward:GTCACCCATCACATGCAGTACATTC	This paper	N/A
E2403B Reverse: ATTAACACGGGGCTTTCCTTTGACC	This paper	N/A
S13a Forward: aagcgatcaaccgacacccaat	This paper	N/A
S13a Reverse: ccgcctgatttgctctgaagaa	This paper	N/A
S13b Forward:gcctttggtgctcgagttagat	This paper	N/A
S13b Reverse:tggacttatatattccaaggga	This paper	N/A
S13c Forward:ttaggctccgtttagtttccaa	This paper	N/A
S13c Reverse:cagtttgggcatcaaacactta	This paper	N/A
S13d Forward:atgcccgcaccagatgagtatt	This paper	N/A
S13d Reverse:ttcttaggcgcaccaaggaaag	This paper	N/A
S13e Forward:ggccaaaaacgtcatatcaaat	This paper	N/A
S13e Reverse:cgtgagttttcctaggagttgg	This paper	N/A
S13f Forward:gtttccgagaaaaatatggttacg	This paper	N/A
S13f Reverse:atatccctgtcctgcttgttgt	This paper	N/A
S13g Forward:gccagcaggtaatagtggaggata	This paper	N/A
S13g Reverse:gctgtcagagttacgtggaacctt	This paper	N/A
S13h Forward: agtccatggcgtaataagcact	This paper	N/A
S13h Reverse:tcccaaggtgacaaactttctt	This paper	N/A
S13i Forward:gataagggggtcatgagcatgtag	This paper	N/A
S13i Reverse: accctcgacgtagttccttaatcc	This paper	N/A
S13j Forward:gagctaagctatacgctcgatg	This paper	N/A
S13j Reverse: gttgtccacgaagttccagaag	This paper	N/A
OlCHR Oligo Forward: ATTCGCTAGGCAGCCCTCTTAT	This paper	N/A
OlCHR Oligo Reverse:CTCCAAAACCCCCAACACTATC	This paper	N/A
Cas9 Forward:CAATAGTAGGTTCGCCTGGATG	This paper	N/A
Cas9 Reverse: TTCGTTGGGGAGGTTCTTG	This paper	N/A
Hygromycin Forward: TTTCTGATCGAAAAGTTCGACAGCGTCT	This paper	N/A
Hygromycin Reverse:GGCAGTTCGGTTTCAGGCAGGTCTTGCAA	This paper	N/A
stop codon in OsCHR745 Forward: ATTTGTTGCTTGCAGGAGTTCC	This paper	N/A
stop codon in OsCHR745 Reverse: CGTCCTGTTTTCTTTCAACGCT	This paper	N/A
35.6 kb insersion in OlCHR homologous region Forward: CATGGAAACCGAATATGCCACA	This paper	N/A
35.6 kb insersion in OlCHR homologous region Reverse: CGATTGCCTAGAGATTCAGGA	This paper	N/A

**Recombinant DNA**		

pZD_OlCHRgRNAU6-Cas9	This study	N/A

**Software and algorithms**		

cutadapt v1.15	https://doi.org/10.14806/ej.17.1.200	N/A
BWA-MEM v0.7.8	https://github.com/lh3/bwa	N/A
picard v2.20.8	https://github.com/broadinstitute/picard	N/A
GATK v3.8	https://kennethjhan.github.io/Genome-Analysis-Tutorial/resource	N/A
tophat2 v2.0.14	https://bioweb.pasteur.fr/packages/pack@tophat@2.0.14	N/A

### Resource availability

#### Lead contact

Further information and requests for resources and reagents should be directed to and will be fulfilled by the lead contact, Yohei Koide (ykoide@agr.hokudai.ac.jp).

#### Materials availability

Plasmids and plant materials newly developed in this study are available from lead contact with a completed Materials Transfer Agreement.

#### Data and code availability

• The raw sequence data are available in the public database (https://www.ddbj.nig.ac.jp/index.html) under the BioProject, PRJDB17754. This paper analyzes existing, publicly available data. These accession numbers for the datasets are listed in the key resources table.
• This paper does not report original code.
• Any additional information required to reanalyze the data reported in this paper is available from the lead contact upon reasonable request.

### Experimental model and study participant details

Wild rice materials were obtained from the National Institute of Genetics supported by the National Bioresource Project, MEXT, Japan, or from the stock of plant breeding laboratory, Hokkaido University, Japan. Rice varieties T65, Nipponbare and A58 were obtained from the stock of plant breeding laboratory, Hokkaido University, Japan. The seeds were sterilized with seed disinfectant Techlead-C flourable (Kumiai-chemical industry) for 10 min and washed with tapped water three times. They are then placed on a Petri dish in 30°C in a dark condition until germinated.

### Method details

#### Development of near-isogenic lines

Taichung 65 is a japonica strain of *Oryza sativa*, and the near-isogenic line (NIL) carrying *wx* as a genetic marker in the Taichung 65 background is simply referred to as “T65” in this study. W1618 is a wild *O. longistaminata* strain originally from Madagascar. For the hybrid sterility locus *S13*, T65 and W1618-derived alleles were named *S13*^*s*^ and *S13*^*l*^, respectively (We named this locus as *S13* because the locus is identical to that previously registered as *S13* by Sano^46^). In the genetic analysis of the *S13* locus, two NILs (T65*A*^*+*^*S13*^*l*^ and T65*S13*^*l*^) were developed through successive backcrossing between T65 as the recurrent parent and W1618 as the donor parent. Although both NILs share the same genotype for the *S13* locus, T65*A*^*+*^*S13*^*l*^ carries the functional allele of the anthocyanin pigmentation gene (*Anthocyanin activator*) located on chromosome 1, which was introgressed along with *S13* from W1618 (*A*^*+*^ stands for wild type allele^63^^,^^64^^,^^65^). In contrast, T65*S13*^*l*^ possesses a nonfunctional allele at the *A* locus from T65. T65*S13*^*l*^ was selected from the segregating population resulting from the cross between T65 and T65*A*^*+*^*S13*^*l*^. The functional gene *A* encodes dihydroflavonol-4-reductase (*DFR*, *Os01g0633500*), and anthocyanin pigmentation served as a visible marker at the seedling stage in this study. In addition to T65, two other *O. sativa* strains (A58 and Nipponbare) were used to identify the *S13* locus. To investigate the allelic condition of the *S13* alleles in African cultivated rice species (*O. glaberrima*), an NIL named T65*A*^*+*^(W025), which carries the functional *A*^*+*^ allele and *S13* from W025 (*O. glaberrima*), was developed and used for the test crossing. Based on the results of the allelic condition investigated, we confirmed that A58 and Nipponbare have the *S13*^*s*^ allele in the *S13* locus (see Results). To examine the distribution of single nucleotide polymorphisms (SNPs) in the *S13* locus among *Oryza* AA genome species, we used 138, 36, and 10 accessions of *O. glaberrima*, *O. barthii*, and *O. meridionalis*, respectively (Table S1). Additionally, a total of 12 accessions without the AA genome within the genus *Oryza* were included in the study (Table S1). Transgenic plants were grown in a greenhouse (natural sunlight with 10 h of supplemented light) at the Research Faculty of Agriculture, Hokkaido University (Sapporo, Japan), whereas all other lines were cultivated under long-day conditions (14 h of light and 10 h of darkness) with short-daylength treatment (10 h of light and 14 h of darkness) if necessary.

#### DNA and RNA extraction

Genomic DNA was extracted from plant leaves using TPS buffer. For whole genome sequencing, the QIAGEN DNeasy® Plant Mini Kit was used. Total RNA from young panicles and other tissues (leaf, young root, leaf sheath, and anther) were extracted using the QIAGEN RNeasy® Plant Mini Kit following the manufacturer’s instructions. Qualification and evaluation of purity of our nucleic acid samples were performed using a Thermo Scientific NanoDrop™ 2000 Spectrophotometer.

#### Cytological analysis of pollen development and fertility phenotyping

For microspore observation and pollen fertility assessment, spikelets were collected from the middle part of the panicles before anthesis. Subsequently, they were fixed in a 1:1:18 solution (formalin: glacial acetic acid: 70% EtOH) and 70% EtOH at room temperature until used for microspore observation. Microspores were stained with acetocarmine and observed under a light microscope (BH-2, Olympus). To estimate pollen fertility, pollens were observed under a light microscope after staining with a potassium iodine solution (I_2_-KI), acetocarmine and haematoxylin. Pollen fertility was quantified as the average percentage of stainable pollens of similar size and roundness in three randomized pictures for each flower, with three flowers per plant.

#### Mapping of the *S13* gene

In rice, anthocyanin pigmentation requires three coloring genes, *C* (*Chromogen for anthocyanin*), *A* (*Anthocyanin activator*), and *P* (*Colored apiculus*).^66^ Oka^67^ showed that the T65 seedling is colorless due to the presence of a nonfunctional allele at the *A* locus. As the *S13*^*l*^ allele of T65*A*^*+*^*S13*^*l*^ possesses the functional color allele *A*^*+*^, DNA markers in the *A* locus (*DFR*) and its flanking region (E2403B) were examined for genotyping. A linkage map was developed by genotyping 300 segregants obtained from the cross between T65*A*^*+*^*S13*^*l*^ and T65 using two DNA markers (*DFR* and E2403B), and phenotyping the pollen fertility. High-resolution mapping was conducted in 2011 segregating plants derived from heterozygote (*S13*^*s*^*/S13*^*l*^) self-pollination using eight additional genetic markers.

#### Next-generation sequencing (NGS) and transcriptome analysis

Genomic DNA extracted from leaves of T65*A*^*+*^*S13*^*l*^ at a young age was sent to Macrogen (https://www.macrogen-japan.co.jp/) for whole genome sequencing using the Illumina sequencing platform. The process involved removing adapters to trim FASTQ raw data using cutadapt (version 1.15). Reads with an average quality of 20 and a minimum length of 30 bp were filtered. Subsequently, the processed data were mapped to the reference genome Nipponbare (IRGSP-1.0) using the BWA mem mapping tool (version 0.7.8). Duplicate reads were removed using the Markduplicate tool (Picard, version 2.20.8), and variant callings of SNPs and InDels were successively identified using a haplotype caller tool in The Genome Analysis Toolkit (GATK, version 3.8).

For RNA-seq analysis, total RNA was extracted from young panicles of T65*A*^*+*^*S13*^*l*^ at the booting stage using the RNeasy Plant Mini Kit (Qiagen). Samples were sequenced using the Illumina sequence platform. After removing and filtering the raw FASTQ files as mentioned above, reads were mapped using tophat2 program (version 2. 0. 14). Read counts were obtained from the resultant BAM file. Computations were partially performed on the NIG supercomputer at the National Institute of Genetics.

#### Preparation of gRNA/Cas9 expressing plasmids

The plasmid pU6gRNA and the single-guide RNA expression vector (pZDgRNA_Cas9ver.2_HPT) were generously provided by Dr. M. Endo from the National Agricultural and Food Research Organization. In general, a single-guide RNA (sgRNA) consisting of 20 nucleotides (N) upstream of the protospacer adjacent motif (PAM) was selected as the target fragment for the candidate gene *OlCHR*. The selected target oligonucleotide sequences are listed in Table S2. The 20-N-long forward sequences (combined with GTTG at the 5′ end) and reverse complementary sequences (combined with AAAC at the 5′ end) of the target sequence were annealed at 95°C for 5 min. Double-stranded target sequences were then cloned into the pU6gRNA vector, which was linearized by BbsI using *Escherichia coli* DH5⍺, following the manufacturer’s protocol (TaKaRa). Subsequently, the synthetic gRNA expression construct (OsU6 promoter:: Target (20N):: gRNA common scaffold:: Poly T termination signal), flanked by PacI and AscI, was inserted into the OsU3-gYSA site in pZDgRNA_Cas9ver.2_HPT. This vector carries the Cas9 expression construct (2xP35S:: OsADH5′UTR:: Os opt. Cas9:: Tpea3A) along with a hygromycin resistance cassette. Additionally, the resulting vector construct, pZDgRNAU6-TargetN_Cas9ver.2_HPT, was cloned using *E. coli* DH5⍺ competent cells following the manufacturer’s protocol (TaKaRa) for transformation.

#### *Agrobacterium tumefaciens*-mediated rice callus transformation and screening of transgenic lines

Rice calli induced from the mature seeds of T65 and T65*A*^*+*^*S13*^*l*^ were sterilized with 70% EtOH and NaClO before placing these explants on callus induction media (N6CI), under light conditions at 25°C for approximately 1 month. Efficient calli were infected through co-cultivation with transformed *A. tumefaciens* EHA105. Subsequently, pZDgRNAU6-TargetN_Cas9ver.2_HPT was introduced into rice callus via *A. tumefaciens*-mediated transformation targeting *OlCHR*. Calli were then regenerated on regeneration media until they reached the plantlet stage, at which point they were transplanted into the transgenic room. The transformation process was based on the method described by Matsumoto and Miyao.^68^ Genomic DNA was extracted from the leaves of transgenic plants using the TPS–chloroform method, amplified using specific primers (Table S3) for *OlCHR*, and processed according to the manufacturer’s protocol (TaKaRa). The amplified DNA fragments were subjected to Sanger sequencing at FASMAC. Detection and determination of the mutated sequence followed to alignment of the sequences obtained from FASMAC with the online database of The Rice Annotation Project (RAP) (https://rapdb.dna.affrc.go.jp/tools/blast) for further analysis.

#### Analysis of allelic distribution of *S13* killer gene

To analyze SNP distribution in *Os01g0636700* in Asian cultivated rice varieties, we used the Rice SNP-Seek Database (https://snp-seek.irri.org/), which contains an SNP dataset of 3,024 varieties. For haplotype analysis, we filtered and exclusively used SNPs located in the CDS of *Os01g0636700*, a homologue of *OlCHR.* For *O. rufipogon* accessions, we used the *Oryza* Genome database (http://viewer.shigen.info/oryzagenome2detail/index.xhtml) to analyze haplotypes of 446 accessions. In the case of other *Oryza* AA genome species and an outgroup of *Oryza* AA genome species, we conducted PCR to amplify a region including the premature stop codon found in *Os01g0636700*, and SNPs were subsequently analyzed by sequencing.

#### Data accessibility

The raw sequence data are available in the public database (https://www.ddbj.nig.ac.jp/index.html) under the BioProject, PRJDB17754.

### Quantification and statistical analysis

The statistical analysis was conducted by using R software (version 4.3.2), Microsoft Excel (version 2402), or Matlab (version 2021b). The statistical details can be found in the figure legends.

## Supplemental information

Document S1. Figures S1–S13 and Tables S2–S5

Table S1. Accessions used, related to Figure 4
