# Supplementary material for: OlCHR, encoding a chromatin remodeling factor, is a killer causing hybrid sterility between rice species Oryza sativa and O. longistaminata
Source: iScience. 2024 Apr 17;27(5):109761. doi: 10.1016/j.isci.2024.109761 (PMC11067373; doi:10.1016/j.isci.2024.109761)
Supplement: Document S1. Figures S1–S13 and Tables S2–S5 [file mmc1.pdf]

iScience, Volume *27*

Supplemental information

OlCHR, encoding a chromatin remodeling factor, is a killer causing hybrid sterility between rice species *Oryza sativa* and *O. longistaminata*

Zin Mar Myint, Yohei Koide, Wakana Takanishi, Tomohito Ikegaya, Choi Kwan, Kiwamu Hikichi, Yoshiki Tokuyama, Shuhei Okada, Kazumitsu Onishi, Ryo Ishikawa, Daisuke Fujita, Yoshiyuki Yamagata, Hideo Matsumura, Yuji Kishima, and Akira Kanazawa

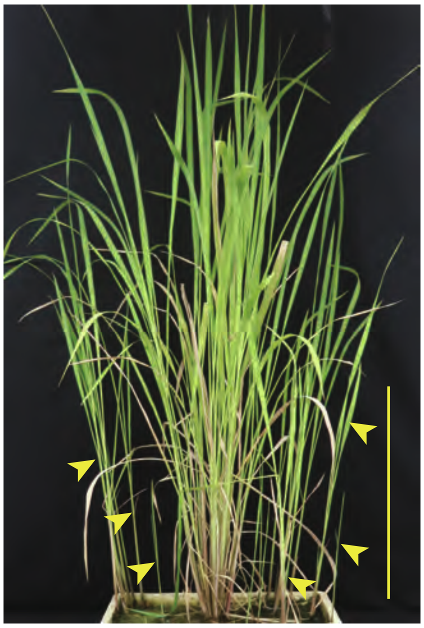

Figure S1.Image of W1618 (*O. longistaminata*), related to Figure 1.
Scale bar = 50 cm. Shoots developed from rhizomes are indicated by arrowheads.

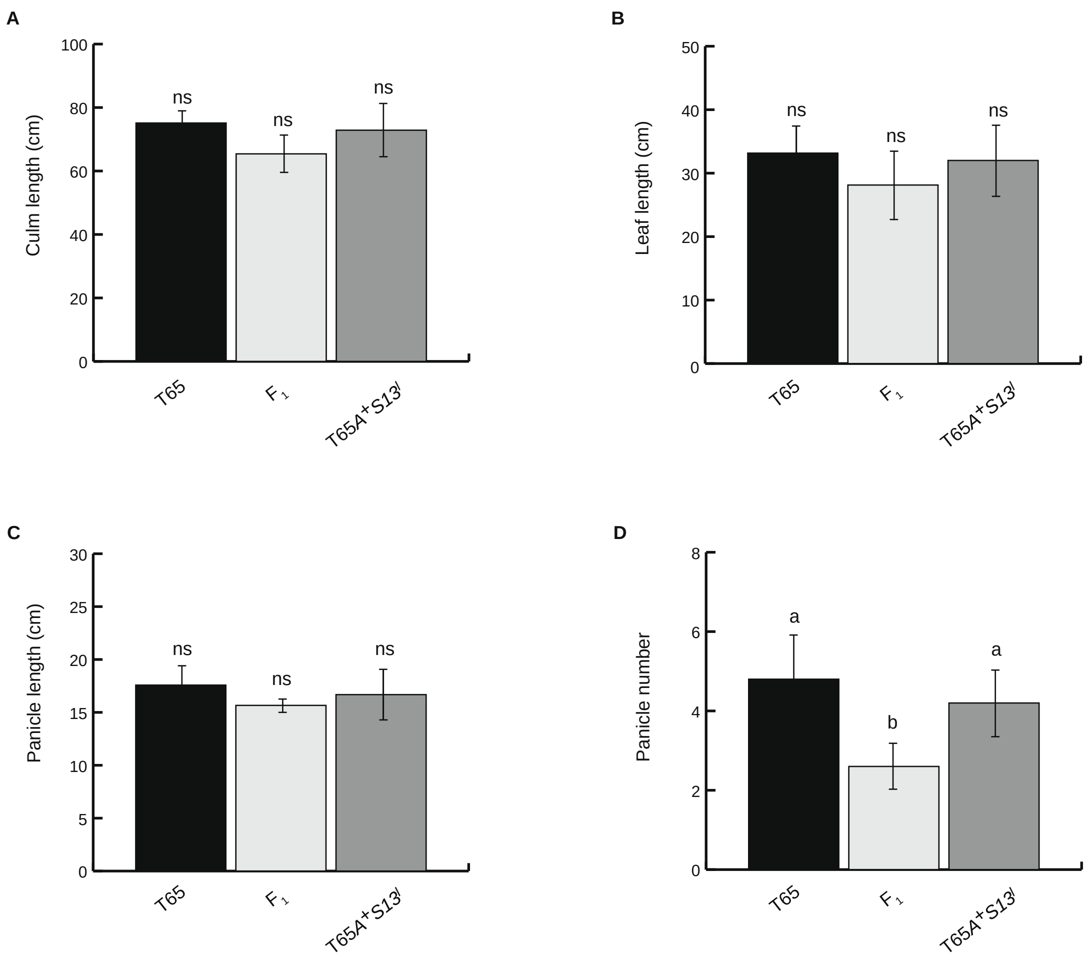

Figure S2. Comparison of agronomic traits in T65, T65$A^{+}S13^{l}$, and their heterozygous F_1, related to Figure 1.
Different letters show significant difference by the Tukey-test at the 5% level of significance.
ns indicates not significant value between varieties.

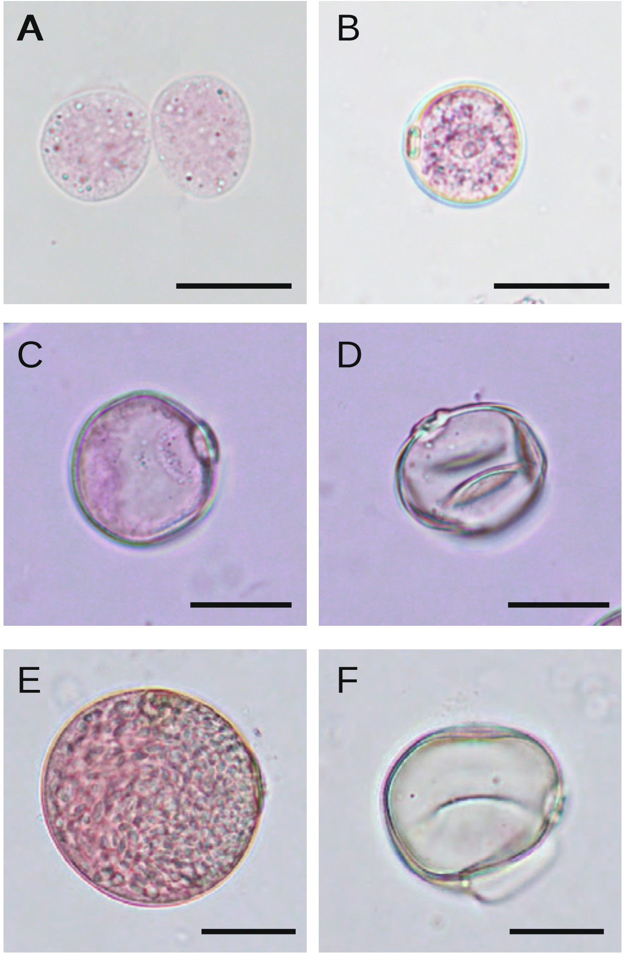

Figure S3. Microspores observed in different developmental stages in heterozygous ($S13^{s}$/$S13^{l}$) plants, related to Figure 1.

(A), (B) Microspores in early one-nucleate and late one-nucleate stages, respectively.

(C) Microspores in two-nuclei stage. (D) Abnormal microspores in two-nuclei stage.

(E) Microspores in three-nuclei stage. (F) Abnormal microspores in three-nuclei stage. Scale bar = 20 μm.

A

Marker genotype[*1]		No. of plants		χ^2 value
		Fertile	Semi-sterile	
DFR	E2403B	(*S13*^{l}/*S13*^{l})	(*S13*^{s}/*S13*^{l})	(1:1)
L	L	162	1	
L	H	0	1	
H	L	0	0	
H	H	1	135	
Total		163	137	2.08^{NS}

B

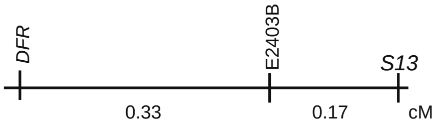

Figure S4. Linkage mapping of the *S13* locus, related to Figure 1.

(A) Frequency of plants with each genotype in the F_2 population.

L and H indicate homozygotes for the *O. longistaminata*-derived allele and heterozygotes, respectively.

(B) Linkage map of the *S13* locus. *DFR* and E2403B indicate the *A* locus and a DNA marker near the *S13* locus, respectively.

```
OsCHR745   GAAGCCGACC GGGAAGTCGC CGCGCCGCCA TCGTGCTCGC CGCCGGTCGC CGTCTCGGAG GAGGAAAAAA GAAAGGAAAA ACTGCAGCGA TGGTACAGAT TAAGGAACTT GGCGAGGGCA
OlCHR      GAAGCCGACC GGGAAGTCGC CGCGCCGCCA TCGTGCTCGC CGCCGGTCGC CGTCTCGGAG GAGGAAAAAA GAAAGGAAAA ACTGCAGCGA TGGTACAGAT TAAGGAACTT GGCGAGGGCA

OsCHR745   GCAGCCATGC TGGGCAAGTT GTTATAAGAG GGCTGCCTAG CGAATTGTCA TATGCTGACC TAGCAGACTA TTTCATTAAA TATGGAAAGA TAGTTGATTT GATTATTATC AGGGCCAAGG
OlCHR      GCAGCCATGC TGGGCAAGTT GTTATAAGAG GGCTGCCTAG CGAATTGTCA TATGCTGACC TAGCAGACTA TTTCATTAAA TATGGAAAGA TAGTTGATTT GATTATTATC AGGGCCAAGG

OsCHR745   GCACAGCCCA GGCAGGGGAT TCTGCTAAAA TTACTTATGC CGATGCTGCC ATTTCTGATA AAATAATCAA GTGCAGGCAT ATTATCAAAG GAAAACATGT TGTAGTTGAT AGAACATTAA
OlCHR      GCACAGCCCA GGCAGGGGAT TCTGCTAAAA TTACTTATGC CGATGCTGCC ATTTCTGATA AAATAATCAA GTGCAGGCAT ATTATCAAAG GAAAACATGT TGTAGTTGAT AGAACATTAA

OsCHR745   TGGAAGACAC CATTCAGTAT AAGGACAAGA AGACAAACCG GAGGATAACT CTGGATGGCC TTCCCTGGAC AGTGTCCAAT GATGATATTG TGCACTTCTT CTCTCCATAT GGAACAGTAG
OlCHR      TGGAAGACAC CATTCAGTAT AAGGACAAGA AGACAAACCG GAGGATAACT CTGGATGGCC TTCCCTGGAC AGTGTCCAAT GATGATATTG TGCACTTCTT CTCTCCATAT GGAACAGTAG

OsCHR745   TTGACCATCA GATCACTCAG AAGGATGAAA ATAAACTTTC TGAAGGATCA GGGTTTGTGC TCTTCAGTTC TGAGCTAGCT GTAATTAAAA TTCTCTCAAA TGGCAACACT GTTAATCTTG
OlCHR      TTGACCATCA GATCACTCAG AAGGATGAAA ATAAACTTTC TGAAGGATCA GGGTTTGTGC TCTTCAGTTC TGAGCTAGCT GTAATTAAAA TTCTCTCAAA TGGCAACACT GTTAATCTTG

OsCHR745   GTGGTGAAAA GGTGTCTATT AATAGATCAG GTGCTTTTGT TATCGCAGCA ACTGGACACC ATATAAAACA CCCCTTTCTC CTTCCTAGTG AGATATTTTC TAGTCTCTTC CCTCATCAAA
OlCHR      GTGGTGAAAA GGTGTCTATT AATAGATCAG GTGCTTTTGT TATCGCAGCA ACTGGACACC ATATAAAACA CCCCTTTCTC CTTCCTAGTG AGATATTTTC TAGTCTCTTC CCTCATCAAA

OsCHR745   AGGATGGGCT TGAATGGCTC TGGAGGCTCC ACTGTGAAAA ATCCGGTGGG GGAATTCTTG CGGATGATAT GGGCCTAGGC AAAACCCGCC AGGCTTCAGC CTTTCTAGCT GGCCTTTTTT
OlCHR      AGGATGGGCT TGAATGGCTC TGGAGGCTCC ACTGTGAAAA ATCCGGTGGG GGAATTCTTG CGGATGATAT GGGCCTAGGC AAAACCCGCC AGGCTTCAGC CTTTCTAGCT GGCCTTTTTT

OsCHR745   ATTCCGATTT GACACAAAGG GTATTGATTG TTGCCCCGGG CACAATCTTG CATCAGTGGA TTGCTGAATT AACAAAAGTT GGTTTTAATG AAGATCTTAT ACATAGCTTC TGGTGTGCCA
OlCHR      ATTCCGATTT GACACAAAGG GTATTGATTG TTGCCCCGGG CACAATCTTG CATCAGTGGA TTGCTGAATT AACAAAAGTT GGTTTTAATG AAGATCTTAT ACATAGCTTC TGGTGTGCCA

OsCHR745   AGACAAGGCA TGATTCTCTA GCGCAGGTGC TAAAGGAAGG TGGTGTTCTG CTTATTACGT ATGATCTGGT GAGGTTGTAT AATGAAGAAC TAAACGGCAT GAGCAGTAAA AGCTCAAAGA
OlCHR      AGACAAGGCA TGATTCTCTA GCGCAGGTGC TAAAGGAAGG TGGTGTTCTG CTTATTACGT ATGATCTGGT GAGGTTGTAT AATGAAGAAC TAAACGGCAT GAGCAGTAAA AGCTCAAAGA

OsCHR745   TGAGAAGAGC TTGTCCTTCA TGGGACTATG TGATTCTTGA TGAGGGTCAC GTGCTGAAGA ACCCAAATAC GAAAAATGCT GCTGCGCTGA AAAGCTTATC TCGTGGGCAA ACAGTTGTCC
OlCHR      TGAGAAGAGC TTGTCCTTCA TGGGACTATG TGATTCTTGA TGAGGGTCAC GTGCTGAAGA ACCCAAATAC GAAAAATGCT GCTGCGCTGA AAAGCTTATC TCGTGGGCAA ACAGTTGTCC

OsCHR745   TCACAGGAAC ACCGGTTCAA AATAACCTTT CGGAATTTCA TTCACTAATG AGTCTTTGCT GTCCTACTGT CTTGGGCTCT CTTGCTGCTT TTGAAAGAGA CTTCTGTAAA CCAATTGACA
OlCHR      TCACAGGAAC ACCGGTTCAA AATAACCTTT CGGAATTTCA TTCACTAATG AGTCTTTGCT GTCCTACTGT CTTGGGCTCT CTTGCTGCTT TTGAAAGAGA CTTCTGTAAA CCAATTGACA

OsCHR745   TGGGAAATGT GCTTGAGGCA ACAACTGAAG TAGTAATGAT ATCTTCTAAA AAGGCCATGG AGTTCCGCAA AATGGTGCGA CCTTATTTTC TTAGGCGCAC CAAGGAAAGT ATTGAAAGCC
OlCHR      TGGGAAATGT GCTTGAGGCA ACAACTGAAG TAGTAATGAT ATCTTCTAAA AAGGCCATGG AGTTCCGCAA AATGGTGCGA CCTTATTTTC TTAGGCGCAC CAAGGAAAGT ATTGAAAGCC

OsCHR745   TTTTACCTAA TAAAGCTGAT CTTGTCATCT GGCTGAAGCT AACGCCATAT CAGATATAAT TATATGAAAC ATTTATGAAG AGCAATTTGA TTGATAAGAC AGTGAAGGGA TCAACATTTG
OlCHR      TTTTACCTAA TAAAGCTGAT CTTGTCATCT GGCTGAAGCT AACGCCATAT CAGATAGAAT TATATGAAAC ATTTATGAAG AGCAATTTGA TTGATAAGAC AGTGAAGGGA TCAACATTTG

OsCHR745   TTGCAACAAT GCTGCTCCAA AAGATATGCA ACCATCCTCA GAACTTGACA GCTGTAGATT CCTGTGAGGA ACAATTAGCG TTGAAAGAAA ACAGGACGTT ACAGGGCATT GTCAAAAAAC
OlCHR      TTGCAACAAT GCTGCTCCAA AAGATATGCA ACCATCCTCA GAACTTGACA GCTGTAGATT CCTGTGAGGA ACAATTAGCG TTGAAAGAAA ACAGGACGTT ACAGGGCATT GTCAAAAAAC

OsCHR745   TTGAAGCTTT GATTGCAAAG AATACCACAA AGACATCAAA TTGTCTCAAG TCATGCAAGC TTACATTTAT ACTGCAATTC AACGAAAAAC TTAAGGAAGA AGGGCACAAG GTGCTAATTT
OlCHR      TTGAAGCTTT GATTGCAAAG AATACCACAA AGACATCAAA TTGTCTCAAG TCATGCAAGC TTACATTTAT ACTGCAATTC AACGAAAAAC TTAAGGAAGA AGGGCACAAG GTGCTAATTT

OsCHR745   TCTCACAGAC GCGCCTAATG CTTGATGAAA TAGAGGAGGC ACTAACCAAC AAGGGAGTAC ATTTTGCTCG TATGGATGGG ACTGTTACAG CTTCTAAAAG AGAAGCTATT ATTAAGGGCT
OlCHR      TCTCACAGAC GCGCCTAATG CTTGATGAAA TAGAGGAGGC ACTAACCAAC AAGGGAGTAC ATTTTGCTCG TATGGATGGG ACTGTTACAG CTTCTAAAAG AGAAGCTATT ATTAAGGGCT

OsCHR745   TTCAAAGTAA AGATGGGCCT CCCATATTTT TAATGACCAC AAAAGTGGGT GGTATAGGTC TAAATTTAAC TAATGCTTCC AGAGTTATCA TCGCTGATCC ATCTTGGAAT CCGAGCCTGG
OlCHR      TTCAAAGTAA AGATGGGCCT CCCATATTTT TAATGACCAC AAAAGTGGGT GGTATAGGTC TAAATTTAAC TAATGCTTCC AGAGTTATCA TCGCTGATCC ATCTTGGAAT CCGAGCCTGG

OsCHR745   ATAATCAATG CGTTGATAGA GTGTACAGAA TTGGACAGGA GAAGAATGTC ATCATCTATA GGCTTATAAC CTCGTGTACT ATTGAGGAAA GAATATATGA AAAACAGGTA TCCAAAGAAG
OlCHR      ATAATCAATG CGTTGATAGA GTGTACAGAA TTGGACAGGA GAAGAATGTC ATCATCTATA GGCTTATAAC CTCGTGTACT ATTGAGGAAA GAATATATGA AAAACAGGTA TCCAAAGAAG

OsCHR745   GGATTTTCAA AGCTGCAACA GAAGAACGTG ATTTCAGGCG CTACATCAAC AAACTGGGTT ACAAAGAATT TTTAAAACTT CCTGAGATGG GTTTCGGAAC ATCCTTATTA CAGAAAAGGC
OlCHR      GGATTTTCAA AGCTGCAACA GAAGAACGTG ATTTCAGGCG CTACATCAAC AAACTGGGTT ACAAAGAATT TTTAAAACTT CCTGAGATGG GTTTCGGAAC ATCCTTATTA CAGAAAAGGC

OsCHR745   TTGAGATAGA GACCATGACA GATAACATTA GGGCAACATT GAAGAGTCAT TTGATATTTT TGAGACAGCA AGGTATAGTA GGCGTATCAG TTCACAACAC ATTGTTTGAG AAAACAATTA
OlCHR      TTGAGATAGA GACCATGACA GATAACATTA GGGCAACATT GAAGAGTCAT TTGATATTTT TGAGACAGCA AGGTATAGTA GGCGTATCAG TTCACAACAC ATTGTTTGAG AAAACAATTA

OsCHR745   ATCTTCCTCC TCAAAAGATT GATATGAAGT ATCTTCCTCC TCAAAAGATT GATATGGGTG ATGAAAATAG AAGTACGGTT GGGCATTCAA GATCTCAGCA CCGCCGATCG CTTTCAGATA
OlCHR      ATCTTCCTCC TCAAAAGATT GATATGAAGT ATCTTCCTCC TCAAAAGATT GATATGGGTG ATGAAAATAG AAGTACGGTT GGGCATTCAA GATCTCAGCA CCGCCGATCG CTTTCAGATA

OsCHR745   GTTGCATAGA TTGGTCTGAG TTAGCAGTGA ATCCAAAAGC AAAACCTGTT AGAAATACAT CATTCAGAGT TGAAAAAGTT GAAGTAGACC GGACTGTGAA AATTGAAGCT AAAAGGGCTG
OlCHR      GTTGCATAGA TTGGTCTGAG TTAGCAGTGA ATCCAAAAGC AAAACCTGTT AGAAATACAT CATTCAGAGT TGAAAAAGTT GAAGTAGACC GGACTGTGAA AATTGAAGCT AAAAGGGCTG

OsCHR745   AGCTAGAATG CAAGACTAAG TTGTATGAAC ATATCAAGGA AGCAGCTCAT GACTCTGGAG CCAATGTTCT TCGACAAATG AAGGCTATTG AAAAAGAGAT TTCTGAACTA ACATCACAAG
OlCHR      AGCTAGAATG CAAGACTAAG TTGTATGAAC ATATCAAGGA AGCAGCTCAT GACTCTGGAG CCAATGTTCT TCGACAAATG AAGGCTATTG AAAAAGAGAT TTCTGAACTA ACATCACAAG

OsCHR745   AAGAGATAGA AGAAAAGAAA AGGAAAGGGG GTTAGCGATG GTCAAACTGC TGAAGAACTC GCGCCAAGTG AAATCTGCCA GTAGACATTT ATTGTTGTCT TCCTTTTGCT TTTTTTTGGG
OlCHR      AAGAGATAGA AGAAAAGAAA AGGAAAGGGG GTTAGCGATG GTCAAACTGC TGAAGAACTC GCGCCAAGTG AAATCTGCCA GTAGACATTT ATTGTTGTCT TCCTTTTGCT TTTTTTTGGG

OsCHR745   GTTCAGGTTT GTATTGCTGT AGAAGCTGGC GATGATGTAG AATTTGTGCC AGTAGAAATT GGTGCTCCAG GATGCAAACA ACAAAGTTCA TTGGTCGATT GACTTTTTAC
OlCHR      GTTCAGGTTT GTATTGCTGT AGAAGCTGGC GATGATGTAG AATTTGTGCC AGTAGAAATT GGTGCTCCAG GATGCAAACA ACAAAGTTCA TTGGTCGATT GACTTTTTAC
```

Figure S5. cDNA alignment of *OsCHR745* and *OlCHR*, related to Figure 2. The translation start site is indicated by a blue box and stop codons are indicated by red boxes. The arrow indicates the SNP causing the premature stop codon in *OsCHR745*.

```
OsCHR745   MVQIKELGEG SSHAGQVVIR GLPSELSYAD LADYFIKYGK IVDLIIIRAK GTAQAGDSAK ITYADAAISD KIIKCRHIIK GKHVVVDRTL MEDTIQYKDK KTNRRITLDG LPWTVSNDDI
OlCHR      MVQIKELGEG SSHAGQVVIR GLPSELSYAD LADYFIKYGK IVDLIIIRAK GTAQAGDSAK ITYADAAISD KIIKCRHIIK GKHVVVDRTL MEDTIQYKDK KTNRRITLDG LPWTVSNDDI
                                                 RNA recognition motif
OsCHR745   VHFFSPYGTV VDHQITQKDE NKLSEGSGFV LFSSELAVIK ILSNGNTVNL GGEKVSINRS GAFVIAATGH HIKHPFLLPS EIFSSLFPHQ KDGLEWLWRL HCEKSGGGIL ADDMGLGKTR
OlCHR      VHFFSPYGTV VDHQITQKDE NKLSEGSGFV LFSSELAVIK ILSNGNTVNL GGEKVSINRS GAFVIAATGH HIKHPFLLPS EIFSSLFPHQ KDGLEWLWRL HCEKSGGGIL ADDMGLGKTR
                             RNA recognition motif
OsCHR745   QASAFLAGLF YSDLTQRVLI VAPGTILHQW IAELTKVGFN EDLIHSFWCA KTRHDSLAQV LKEGGVLLIT YDLVRLYNEE LNGMSSKSSK MRRACPSWDY VILDEGHVLK NPNTKNAAAL
OlCHR      QASAFLAGLF YSDLTQRVLI VAPGTILHQW IAELTKVGFN EDLIHSFWCA KTRHDSLAQV LKEGGVLLIT YDLVRLYNEE LNGMSSKSSK MRRACPSWDY VILDEGHVLK NPNTKNAAAL
                                                         Snf2, N-terminal
OsCHR745   KSLSRGQTVV LTGTPVQNNL SEFHSLMSLC CPTVLGSLAA FERDFCKPID MGNVLEATTE VVMISSKKAM EFRKMVRPYF LRRTKESIES LLPNKADLVI WLKLTPYQI* ----------
OlCHR      KSLSRGQTVV LTGTPVQNNL SEFHSLMSLC CPTVLGSLAA FERDFCKPID MGNVLEATTE VVMISSKKAM EFRKMVRPYF LRRTKESIES LLPNKADLVI WLKLTPYQIE LYETFMKSNL

OsCHR745   ---------- ---------- ---------- ---------- ---------- ---------- ---------- ---------- ---------- ---------- ---------- ----------
OlCHR      IDKTVKGSTF VATMLLQKIC NHPQNLTAVD SCEEQLALKE NRTLQGIVKK LEALIAKNTT KTSNCLKSCK LTFILQFNEK LKEEGHKVLI FSQTRLMLDE IEEALTNKGV HFARMDGTVT

OsCHR745   ---------- ---------- ---------- ---------- ---------- ---------- ---------- ---------- ---------- ---------- ---------- ----------
OlCHR      ASKREAIIKG FQSKDGPPIF LMTTKVGGIG LNLTNASRVI IADPSWNPSL DNQCVDRVYR IGQEKNVIIY RLITSCTIEE RIYEKQVSKE GIFKAATEER DFRRYINKLG YKEFLKLPEM
                                                         Helicase, C-terminal
OsCHR745   ---------- ---------- ---------- ---------- ---------- ---------- ---------- ---------- ---------- ---------- ---------- ----------
OlCHR      GFGTSLLQKR LEIETMTDNI RATLKSHLIF LRQQGIVGVS VHNTLFEKTI NLPPQKIDMK YLPPQKIDMG DENRSTVGHS RSQHRRSLSD SCIDWSELAV NPKAKPVRNT SFRVEKVEVD

OsCHR745   ---------- ---------- ---------- ---------- ---------- ---------- --
OlCHR      RTVKIEAKRA ELECKTKLYE HIKEAAHDSG ANVLRQMKAI EKEISELTSQ EEIEEKKRKG G*
```

Figure S6. Amino acid alignment of *OsCHR745* and *OlCHR*, related to Figure 2.

The positions of predicted domains (RNA recognition motif, Snf2, and helicase) are shown below the sequences.

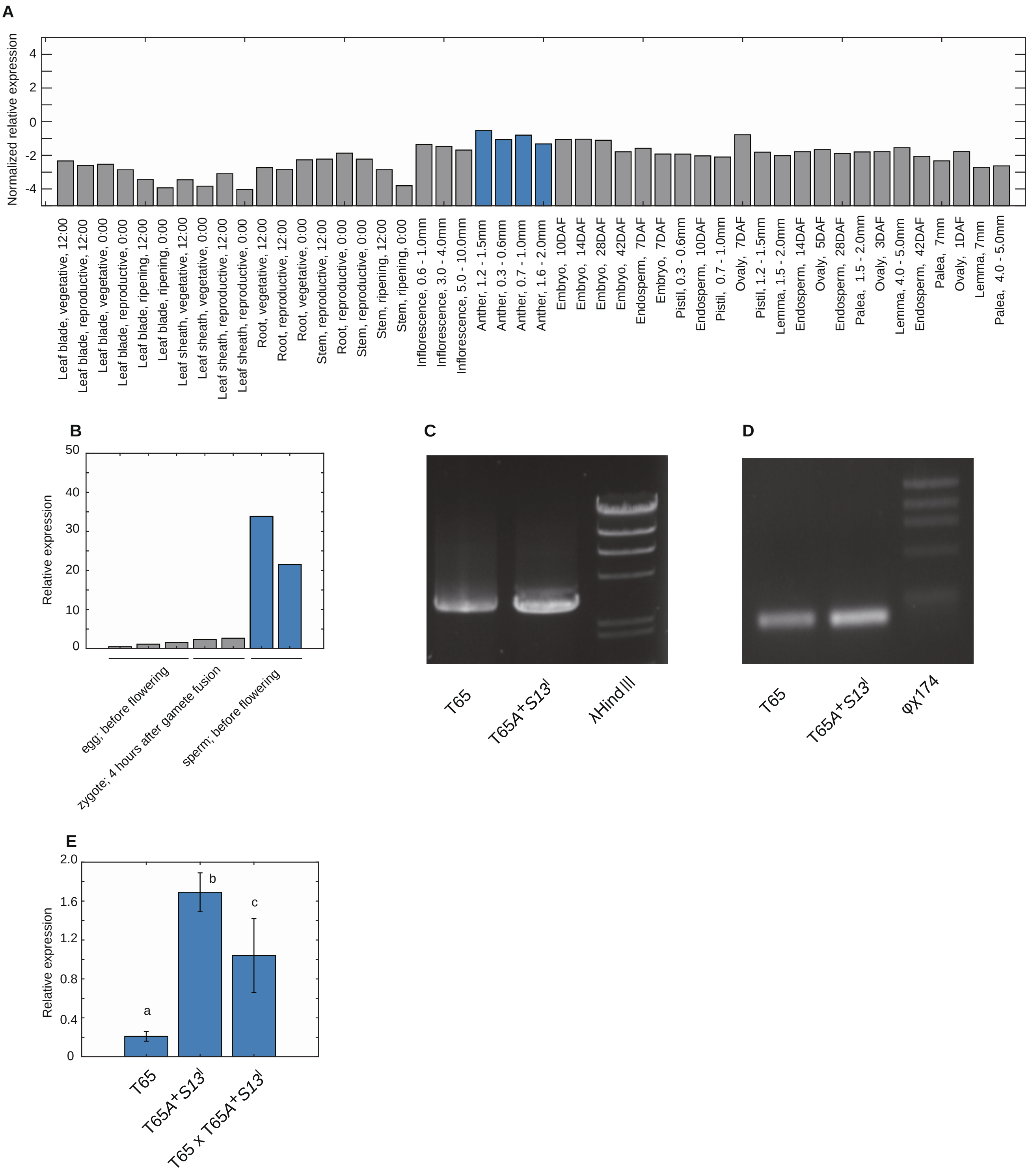

Figure S7. *OsCHR745* expression, related to Figure 2.

(A) *OsCHR745* (*Os01g0636700*) expression in various tissues in rice as shown in RiceXpro database.

(B) *OsCHR745* (*Os01g0636700*) expression in egg and sperm cells as reported

by Rahman et al. (2019) in the Rice Annotation Project database (https://rapdb.dna.affrc.go.jp/index.html).

(C) and (D) RT-PCR using RNA extracted from anthers before flowering. (C) *OsCHR745* and *OlCHR*. (D) *Actin*.

(E) *OsCHR745* (*Os01g0636700*) using qRT-PCR. Different letters show significant difference by the Tukey-test at the 5% level of significance.

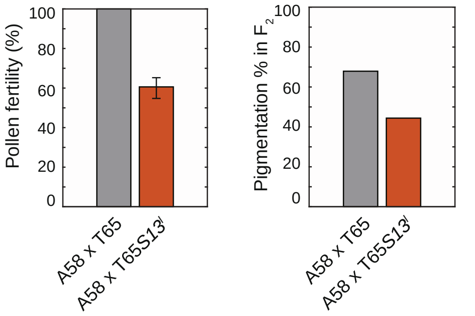

Figure S8. Test cross for identifying the allele in the *S13* locus in A58, related to Figure 2.

Pollen fertility (left) and frequency of pigmented plants in the F_2 population (right).

Seedling pigmentation in the F_2 population was used to check distortion of the *A* (*Anthocyanin activator*) locus linked with the *S13* locus.

Orange bars indicate semi-sterile pollen and segregation ratio distorted cross combination.

	25524921	25524980	25525014	25525015	25525124	25525158	25525323	25525327	25525522	25525666	25525667	25526259	25526298	25527150	25527162	25527287	25527540	25527578	25527791	25527848	25528224	25528377	25528425	25528426	25529067	25529201	25529257	25529313	25529428	25529616	25529883	25530554	25530556	
	22	22	22	22	21	21	20	20	19	18	18	16	16	15	15	14	13	13	12	12	10	9	9	9	8	7	7	7	6	6	5	3	3	
IRGSP1.0	G	T	G	C	G	C	C	C	T	C	G	C	T	G	C	C	G	C	T	A	G	G	G	C	G	T	G	C	G	C	C	G	A	
SNP effect	NS	S	NS	NS	NS	S	ST	NS	S	NS	NS	NS	NS	NS	NS	NS	NS	NS	NS	SL	NS	NS	NS	NS	NS	NS	S	NS	S	NS	ST	NS	S	o. of varieti
Hap. 1	A	T	G	C	G	C	C	C	T	C	A	C	T	G	C	C	G	C	T	C	G	G	G	C	G	T	G	C	G	C	C	G	A	1
Hap. 2	A	T	G	C	G	C	C	C	T	C	G	C	T	G	C	C	G	C	T	C	A	G	G	C	G	T	G	C	G	C	C	G	A	2
Hap. 3	A	T	G	C	G	C	C	C	T	C	G	C	T	G	C	C	G	C	T	C	G	A	G	C	G	T	G	C	G	C	C	G	A	1
Hap. 4	A	T	G	C	G	C	C	C	T	C	G	C	T	G	C	C	G	C	T	C	G	G	G	C	A	T	G	C	G	C	C	G	A	1
Hap. 5	A	T	G	C	G	C	C	C	T	C	G	C	T	G	C	C	G	C	T	C	G	G	G	C	G	C	G	C	G	C	C	G	A	1
Hap. 6	A	T	G	C	G	C	C	C	T	C	G	C	T	G	C	C	G	C	T	C	G	G	G	C	G	T	G	C	G	C	C	G	A	1294
Hap. 7	A	T	G	C	G	C	C	C	T	C	G	C	T	G	C	C	G	C	T	C	G	G	G	C	G	T	G	C	T	C	C	G	A	1
Hap. 8	A	T	G	C	G	C	C	C	T	C	G	C	T	G	C	C	G	C	T	C	G	G	G	C	G	T	G	T	G	C	C	G	A	6
Hap. 9	A	T	G	C	G	C	C	C	T	T	G	C	T	G	C	C	G	C	T	C	G	G	G	C	G	T	G	C	G	C	C	G	A	1
Hap. 10	A	T	G	C	G	C	C	T	T	C	G	C	T	G	C	C	G	C	T	C	G	G	G	C	G	T	G	C	G	C	C	G	A	3
Hap. 11	A	T	G	C	G	T	C	C	T	C	G	C	T	G	C	C	G	C	T	C	G	G	G	C	G	T	G	C	G	C	C	G	A	1
Hap. 12	G	C	G	C	G	C	C	C	T	C	G	C	T	G	C	C	G	C	T	C	G	G	G	C	G	T	G	C	G	C	C	G	A	3
Hap. 13	G	T	G	C	A	C	C	C	T	C	G	C	T	G	C	C	G	C	T	A	G	G	G	C	G	T	G	C	G	C	C	G	A	6
Hap. 14	G	T	G	C	G	C	A	C	T	C	G	C	T	G	C	C	G	C	T	C	G	G	G	C	G	T	G	C	G	C	C	G	A	6
Hap. 15	G	T	G	C	G	C	A	C	T	C	G	C	T	G	T	C	G	C	T	C	G	G	G	C	G	T	G	C	G	C	C	G	A	1
Hap. 16	G	T	G	C	G	C	C	C	C	C	G	C	T	G	C	C	G	C	T	A	G	G	G	C	G	T	G	C	G	C	C	G	A	1
Hap. 17	G	T	G	C	G	C	C	C	T	C	G	C	C	G	C	C	G	C	T	A	G	G	G	C	G	T	G	C	G	C	C	G	A	1
Hap. 18	G	T	G	C	G	C	C	C	T	C	G	C	T	A	C	C	G	C	T	C	G	G	G	C	G	T	G	C	G	C	C	G	A	1
Hap. 19	G	T	G	C	G	C	C	C	T	C	G	C	T	G	C	A	G	C	T	C	G	G	G	C	G	T	G	C	G	C	C	G	A	4
Hap. 20	G	T	G	C	G	C	C	C	T	C	G	C	T	G	C	C	A	C	T	C	G	G	G	C	G	T	G	C	G	C	C	G	A	1
Hap. 21	G	T	G	C	G	C	C	C	T	C	G	C	T	G	C	C	G	A	T	C	G	G	G	C	G	T	G	C	G	C	C	G	A	1
Hap. 22	G	T	G	C	G	C	C	C	T	C	G	C	T	G	C	C	G	C	A	A	G	G	G	C	G	T	G	C	G	C	C	G	A	1
Hap. 23	G	T	G	C	G	C	C	C	T	C	G	C	T	G	C	C	G	C	T	A	G	G	G	C	G	T	A	C	G	C	C	G	A	17
Hap. 24	G	T	G	C	G	C	C	C	T	C	G	C	T	G	C	C	G	C	T	A	G	G	G	C	G	T	G	C	G	C	C	G	A	597
Hap. 25	G	T	G	C	G	C	C	C	T	C	G	C	T	G	C	C	G	C	T	A	G	G	G	C	G	T	G	C	G	C	C	G	G	1
Hap. 26	G	T	G	C	G	C	C	C	T	C	G	C	T	G	C	C	G	C	T	C	G	G	A	C	G	T	G	C	G	C	C	G	A	1
Hap. 27	G	T	G	C	G	C	C	C	T	C	G	C	T	G	C	C	G	C	T	C	G	G	G	C	G	T	G	C	G	A	C	G	A	1
Hap. 28	G	T	G	C	G	C	C	C	T	C	G	C	T	G	C	C	G	C	T	C	G	G	G	C	G	T	G	C	G	C	A	G	A	32
Hap. 29	G	T	G	C	G	C	C	C	T	C	G	C	T	G	C	C	G	C	T	C	G	G	G	C	G	T	G	C	G	C	C	A	A	1
Hap. 30	G	T	G	C	G	C	C	C	T	C	G	C	T	G	C	C	G	C	T	C	G	G	G	C	G	T	G	C	G	C	C	G	A	563
Hap. 31	G	T	G	C	G	C	C	C	T	C	G	C	T	G	C	C	G	C	T	C	G	G	G	T	G	T	G	C	G	C	C	G	A	1
Hap. 32	G	T	G	C	G	C	C	C	T	C	G	T	T	G	C	C	G	C	T	A	G	G	G	C	G	T	G	C	G	C	C	G	A	5
Hap. 33	G	T	T	G	G	C	C	C	T	C	G	C	T	G	C	C	G	C	T	C	G	G	G	C	G	T	G	C	G	C	C	G	A	1

Figure S9. Haplotypes classified by SNPs in the coding region of *OsCHR745* using the rice 3k database, related to Figure 3.

The number of exons where SNPs were located, reference allele, and expected SNP effect are shown below the chromosomal position.

In the SNP table, nonreference type variants are shown in orange and variants causing the premature stop codon are shown in blue.

		25524921	25525323	25525666	25527848	25529257	25529313	25529883
WRC01_Nipponbare WRC01	JP	G	C	C	A	G	C	C
WRC02_Kasalath WRC02	AUS	G	C	C	C	G	C	C
WRC03_BeiKhe WRC03	IND	G	C	C	C	G	C	C
WRC04_Jena035 WRC04	IND	G	C	C	-	-	-	-
WRC05_Naba WRC05	IND	G	C	C	-	-	-	-
WRC06_PuluikArang WRC06	IND	A	C	C	C	G	C	C
WRC07_Davao1 WRC07	IND	G	C	C	C	G	C	A
WRC09_RyouSuisanKoumai WRC09	IND	G	C	C	A	G	C	C
WRC10_QiuZaoZhong WRC10	IND	A	C	C	C	G	C	C
WRC11_Jinguoyin WRC11	IND	A	C	C	C	G	C	C
WRC12_DaHongGu WRC12	IND	A	C	C	C	G	C	C
WRC13_Asu WRC13	IND	A	C	C	C	G	C	C
WRC14_IR58 WRC14	IND	A	C	C	C	G	C	C
WRC15_Co13 WRC15	IND	G	C	C	C	G	C	C
WRC16_VaryFutsi WRC16	IND	G/A	C	C	C	G	C	C
WRC17_Keiboba WRC17	IND	G	C	C	C	G	C	A
WRC18_Qingyu WRC18	IND	A	C	C	C	G	C	C
WRC19_DengPaoZhai WRC19	IND	A	C	C	C	G	C	C
WRC20_Tadukan WRC20	JP	A	C	C	C	G	C	C
WRC21_ShweNangGyi WRC21	IND	A	C	C	C	G	C	C
WRC22_Calotoc WRC22	JP	A	C	C	C	G	C	C
WRC23_Lebed WRC23	JP	G	C	C	C	G	C	C
WRC24_Pinulupot1 WRC24	JP	G	C	C	C	G	C	C
WRC25_Muha WRC25	AUS	G	C	C	C	G	C	C
WRC26_Jhona2 WRC26	AUS	G	C	C	A	G	C	C
WRC27_Nepal8 WRC27	AUS	G	C	C	C	G	C	C
WRC28_Jarjan WRC28	AUS	G	C	C	C	G	C	C
WRC29_KaloDhan WRC29	AUS	G	C	C	-	-	-	-
WRC30_AnjanaDhan WRC30	IND	G	C	C	C	G	C	C
WRC31_Shoni WRC31	AUS	G	C	C	C	G	C	C
WRC32_Tupa121-3 WRC32	IND	G	C	C	-	-	-	-
WRC33_Surjamukhi WRC33	AUS	G	C	C	C	G	C	C
WRC34_ARC7291 WRC34	AUS	G	C	C	C	G	C	C
WRC35_ARC5955 WRC35	AUS	G	C	C	C	G	C	C
WRC36_Ratul WRC36	AUS	G	C	C	C	G	C	C
WRC37_ARC7047 WRC37	AUS	G	C	C	C	G	C	C
WRC38_ARC11094 WRC38	AUS	G	C	C	C	G	C	C
WRC39_BadariDhan WRC39	IND	G	C	C	C	G	C	C
WRC40_Nepal555 WRC40	AUS	G	C	C	C	G	C	C
WRC41_Kaluheenati WRC41	AUS	G	C	C	C	G	C	C
WRC42_LocalBasmati WRC42	AUS	G	C	C	C	G	C	C
WRC43_Dianyu1 WRC43	JP	G	C	C	A	G	C	C
WRC44_Basilanon WRC44	TRJ	G	C	C	C	G	C	C
WRC45_Masho WRC45	TRJ	G	C	C	C	G	C	C
WRC46_KhaoNok WRC46	JP	G	C	C	A	G	C	C
WRC47_Jaguary WRC47	JP	G	C	C	C	G	C	C
WRC48_KhauMacKho WRC48	TRJ	G	C	C	A	A	C	C
WRC49_PadiPerak WRC49	TRJ	G	C	C	C	G	C	C
WRC50_Rexmont WRC50	JP	G	C	C	C	G	C	C
WRC51_Urasan1 WRC51	JP	G	C	C	A	G	C	C
WRC52_KhauTanChiem WRC52	JP	G	C	C	A	G	C	C
WRC53_Tima WRC53	TRJ	G	C	C	A	G	C	C
WRC55_Tupa729 WRC55	JP	G	A	C	C	G	C	C
WRC57_Milyang23 WRC57	IND	A	C	C	C	G	C	C
WRC58_NeangMenh WRC58	IND	A	C	C	C	G	C	C
WRC59_NeangPhtong WRC59	IND	A	C	C	C	G	C	C
WRC60_Hakphaynhay WRC60	IND	A	C	C	C	G	C	C
WRC61_RadinGoiSesat WRC61	IND	G/A	C	C	A/C	G	C/T	C
WRC62_Kemasin WRC62	IND	A	C	C	C	G	C	C
WRC63_Bleiyo WRC63	IND	A	C	C	C	G	C	C
WRC64_PadiKuning WRC64	IND	G	C	C	C	G	C	C
WRC65_Rambhog WRC65	IND	A	C	C	C	G	C	C
WRC66_Bingala WRC66	IND	G	C	C	C	G	C	A
WRC67_Phulba WRC67	JP	G	C	C	A	G	C	C
WRC68_KhaoNamJen WRC68	TRJ	G	C	C	A	G	C	C
WRC97_ChinGalay WRC97	IND	G	C	C	A	G	C	C
WRC98_Deejiaohualuo WRC98	IND	A	C	C	C	G	C	C
WRC99_HongCheuhZai WRC99	IND	G	C	C	C	G	C	A
WRC100_Vandaran WRC100	IND	A	C	C	C	G	C	C
JRC01_Gaisen_Mochi JRC01	TRJ	A	C	C	C	G	C	C
JRC03_Hinode JRC03	TRJ	A	C	C	C	G	C	C
JRC04_Senshou JRC04	TRJ	A	C	C	C	G	C	C
JRC05_Yamada_Bake JRC05	TRJ	G	C	C	C	G	C	C
JRC06_Kaneko_B JRC06	TRJ	G	C	C	C	G	C	C
JRC07_Iruma_Nishiki JRC07	TRJ	G	C	C	A	G	C	C
JRC08_Okka_Modoshi JRC08	TRJ	G	C	C	A	G	C	C
JRC10_Hirayama JRC10	TRJ	A	C	C	A	G	C	C
JRC11_Kahei JRC11	TRJ	G	C	C	A	G	C	C
JRC12_Oiran JRC12	TRJ	G	C	C	A	G	C	C
JRC13_Bouzu_Mochi JRC13	TRJ	G	C	C	A	G	C	C
JRC14_Meguro_Mochi JRC14	TRJ	A	C	C	C	G	C	C
JRC17_Akage JRC17	JP	G	C	C	A	G	C	C
JRC18_Hassokuho JRC18	JP	G	C	C	A	G	C	C
JRC19_Wataribune JRC19	JP	G	C	C	A	G	C	C
JRC20_Hosogara JRC20	JP	G	C	C	A	G	C	C
JRC21_Akamai_Kouchi JRC21	IND	G	C	C	C	G	C	A
JRC22_Mansaku JRC22	JP	G	C	C	A	G	C	C
JRC23_Ishijiro JRC23	JP	G	C	C	A	G	C	C
JRC24_Joushuu JRC24	JP	G	C	C	A	G	C	C
JRC25_Dango JRC25	JP	G	C	C	A	G	C	C
JRC26_Aikoku JRC26	JP	G	C	C	A	G	C	C
JRC27_Ginbouzu JRC27	JP	G	C	C	A	G	C	C
JRC28_Shinriki_Mochi JRC28	JP	G	C	C	A	G	C	C
JRC29_Shichimenchou_Mochi JRC29	JP	G	C	C	A	G	C	C
JRC30_Morita_Wase JRC30	JP	G	C	C	A	G	C	C
JRC31_Kameji JRC31	JP	G	C	C	A	G	C	C
JRC32_Omachi JRC32	JP	G	C	C	A	G	C	C
JRC33_Shinriki JRC33	JP	G	C	C	A	G	C	C
JRC34_Kyoutoasahi JRC34	JP	G	C	C	A	G	C	C
JRC35_Kabashiko JRC35	JP	G	C	C	A	G	C	C
JRC36_Sekiyama JRC36	JP	G	C	C	A	G	C	C
JRC37_Shinyamadaho_2 JRC37	JP	G	C	C	A	G	C	C
JRC38_Nagoya_Shiro JRC38	JP	G	C	T	A	G	C	C
JRC39_Shiroine_Kemomi JRC39	JP	G	C	C	A	G	C	C
JRC40_Akamai_Nagasaki JRC40	JP	G	C	C	A	G	C	C
JRC41_Akamai_Tokushima JRC41	IND	A	C	C	C	G	C	C
JRC42_Touboshi JRC42	IND	A	C	C	C	G	C	C
JRC43_Akamai_Kantou JRC43	IND	G	C	C	C	G	C	A
JRC44_Karahoushi JRC44	IND	G	C	C	A	G	C	C
JRC45_Hiyadachitou JRC45	JP	G	C	C	A	G	C	C
JRC46_Fukoku JRC46	JP	G	C	C	A	G	C	C
JRC47_Okabo JRC47	JP	A	C	C	C	G	C	C
JRC48_Hakamuri_Yokoyama JRC48	JP	G	C	C	A	G	C	C
JRC49_Rikutou_Rikuu_2 JRC49	JP	G	C	C	A	G	C	C
JRC50_Himenomochi JRC50	JP	G	C	C	A	G	C	C
JRC51_Shinshiuu JRC51	JP	G	C	C	A	G	C	C
JRC52_Aichiasahi JRC52	JP	G	C	C	A	G	C	C
JRC53_Raiden JRC53	JP	G	C	C	A	G	C	C
JRC54_Houmanshinden_Ine JRC54	JP	G	C	C	A	G	C	C

Figure S10. Genotypes of SNP loci in the coding region of *OsCHR745* using the World Rice Core Collection (WRC) and Japan Rice Core Collection (JRC) databases of the research center of genetic resources, NARO (National Agriculture and Food Research Organization), Japan, related to Figure 3.
The chromosomal location of SNPs were shown above the data of genotypes. JP, AUS, IND and TRJ indicate temperate japonica, aus, indica, tropical japonica groups, respectively. In the SNP table, nonreference type variants without causing premature stop codon are shown in orange and variants causing the premature stop codon are shown in blue.

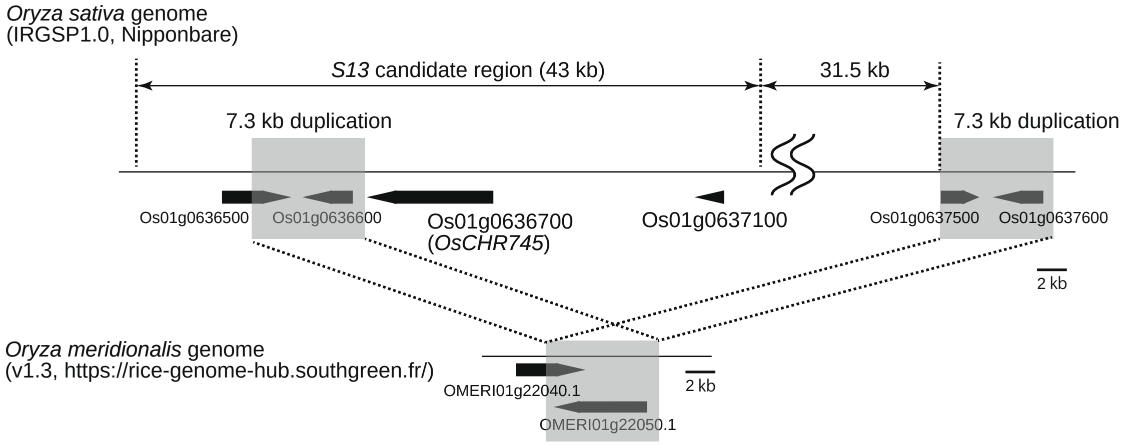

Figure S12. Structural variation in the *S13* locus observed between *O. sativa* and *O. meridionalis*, related to Figure 4.

The *S13* candidate region (43 kb) and its downstream (48.8 kb) are shown.

Gray boxes indicate duplicated regions. In *O. meridionalis,* about 58.5 kb region between the duplicated regions were deleted.

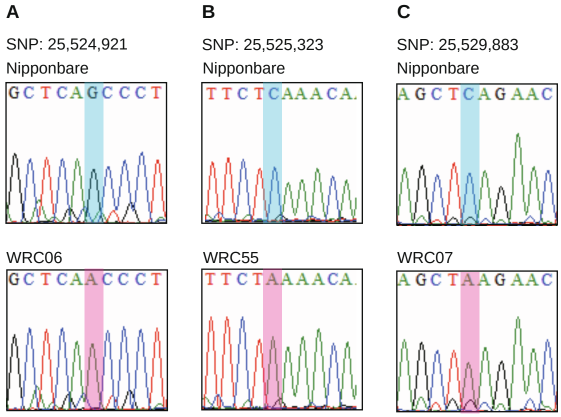

Figure S11. The presence of SNPs confirmed by sequencing electropherogram of loci in the coding region of *OsCHR745* using the World Rice Core Collection (WRC) of the research center of genetic resources, NARO (National Agriculture and Food Research Organization), Japan, related to Figure 3.

(A) The presence of a widespread SNP in the position of 25,524,921 bp in chromosome 1.

(B), (C) The presence of SNPs (in 25,525,323 and 25,529,883, respectively) causing premature stop codons in *OlCHR*.

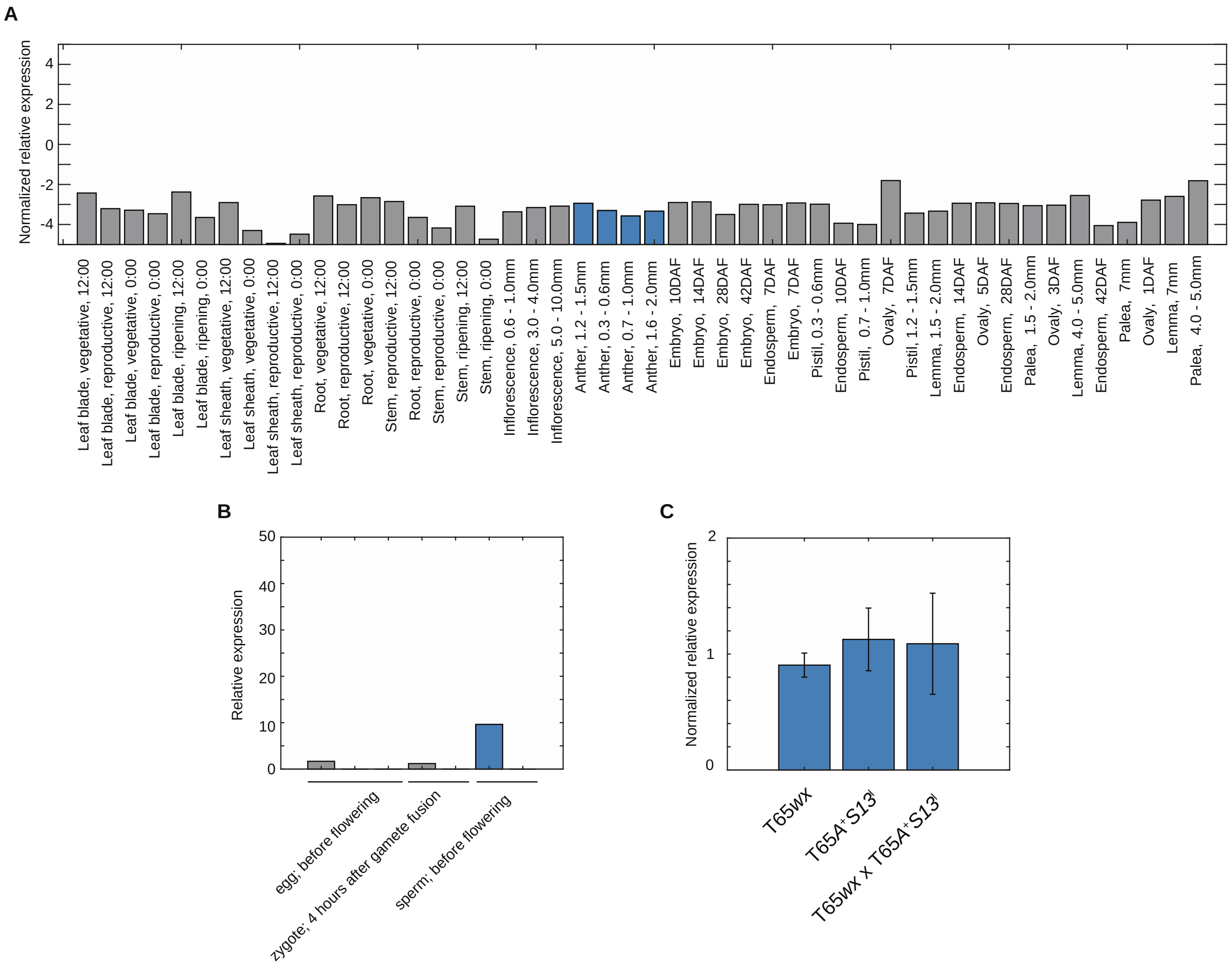

Figure S13.*Os01g0637100* expression, related to Figure 1.

(A) *Os01g0637100* expression in various tissues in rice as shown in RiceXpro database.

(B) *Os01g0637100* expression in egg and sperm cells as reported by Rahman et al. (2019) in the Rice Annotation Project database (https://rapdb.dna.affrc.go.jp/index.html).

(C) Normalized relative expression of *Os01g0637100* calculated from microarray data normalized by median relative expression values.

Table S2. Primers to detect mutation for the candidate gene, *OlCHR*, related to Figure 2.

Target gene	Primer pairs		Target sequence
OlCHR	Fw:	5' ATTCGCTAGGCAGCCCTCTTAT 3'	5' CGCCAAGTTCCTTAATCTGT 3'
	Rv:	5' CTCCAAAACCCCCAACACTATC 3'	

Table S3. Primers used in this study, related to Figure 1.

Target		Primer pairs	Purpose
OlCHR	Fw:	5' ATTCGCTAGGCAGCCCTCTTAT 3'	Mutagenesis
	Rv:	5' CTCCAAAACCCCCAACACTATC 3'	
Cas9	Fw:	5' CAATAGTAGGTTCGCCTGGATG 3'	Mutagenesis
	Rv:	5' TTCGTTGGGGAGGTTCTTG 3'	
Hygromycin	Fw:	5' TTTCTGATCGAAAAGTTCGACAGCGTCT 3'	Mutagenesis
	Rv:	5' GGCAGTTCGGTTTCAGGCAGGTCTTGCAA 3'	
stop codon in *OsCHR745*	Fw:	5' ATTTGTTGCTTGCAGGAGTTCC 3'	Haplotype analysis
	Rv:	5' CGTCCTGTTTTCTTTCAACGCT 3'	
35.6 kb insersion in *OlCHR* homologous region	Fw:	5' CATGGAAACCGAATATGCCACA 3'	Presence of *OlCHR* homologue
	Rv:	5' CGATTGCCTAGAGATTCAGGA 3'	
DFR	Fw:	5' aggtgcacgtagctcaaaccta 3'	Mapping
	Rv:	5' agctaccttgcacttggtgatg 3'	
E2403B	Fw:	5' GTCACCCATCACATGCAGTACATTC 3'	Mapping
	Rv:	5' ATTAACACGGGGCTTTCCTTTGACC 3'	
S13-a	Fw:	5' aagcgatcaaccgacacccaat 3'	Mapping
	Rv:	5' ccgcctgatttgctctgaagaa 3'	
S13-b	Fw:	5' gcctttggtgctcgagttagat 3'	Mapping
	Rv:	5' tggacttatatattccaaggga 3'	
S13-c	Fw:	5' ttaggctccgtttagtttccaa 3'	Mapping
	Rv:	5' cagtttgggcatcaaacactta 3'	
S13-d	Fw:	5' atgcccgcaccagatgagtatt 3'	Mapping
	Rv:	5' ttcttaggcgcaccaaggaaag 3'	
S13-e	Fw:	5' ggccaaaaacgtcatatcaaat 3'	Mapping
	Rv:	5' cgtgagttttcctaggagttgg 3'	
S13-f	Fw:	5' gtttccgagaaaaatatggttacg 3'	Mapping
	Rv:	5' atatccctgtcctgcttgttgt 3'	
S13-g	Fw:	5' gccagcaggtaatagtggaggata 3'	Mapping
	Rv:	5' gctgtcagagttacgtggaacctt 3'	
S13-h	Fw:	5' agtccatggcgtaataagcact 3'	Mapping
	Rv:	5' tcccaaggtgacaaactttctt 3'	
S13-i	Fw:	5' gataagggggtcatgagcatgtag 3'	Mapping
	Rv:	5' accctcgacgtagttccttaatcc 3'	
S13-j	Fw:	5' gagctaagctatacgctcgatg 3'	Mapping
	Rv:	5' gttgtccacgaagttccagaag 3'	

Table S4. Segregation patterns for pollen-semi-sterility observed in plants during backcrosses between T65 and W1618, related to Figure 1.

Cross combinations		Generation	Average of pollen fertility of semi-st. plant (%)	No. of plants								
				Pollen fertility								
Female	Male			<25	35	45	55	65	75	85	95	Total
T65	W1618	F_1	2.3	3								3
Semi-st.	T65	B_1F_1	12	2	1	1						5
		B_2F_1	38		2	1	2					5
		B_3F_1	47		1	2	1		1	2		7

Semi-st. indicates pollen semi-sterility plants.

T65 was used as the reccurent parent.

Table S5. Frequencies of abnormal microspores during the microsporgenesis in the heterozygote of *S13*^{s}/*S13*^{1}, related to Figure 1.

Stage	No. of microspores			Ratio	X^2
	Normal	Abnormal	Total		
One-nucleus	100	0	100	1 : 0	-
Two-nuclei	56	44	100	1 : 1	1.44 ^{ns}
Mature	199	201	400	1 : 1	0.01 ^{ns}

^{ns} shows non-significance (X^2 test)

Supplemental references

Rahman, M.H., Toda, E., Kobayashi, M., Kudo, T., Koshimizu, S., Takahara, M., Iwami, M., Watanabe, Y., Sekimoto, H., Yano, K. and Okamoto, T. (2019). Expression of genes from paternal alleles in rice zygotes and involvement of *OsASGR-BBML1* in initiation of zygotic development. Plant Cell Physiol. *60*(4), 725-737. 10.1093/pcp/pcz030.
